# AURKB/CDC37 complex promotes clear cell renal cell carcinoma progression via phosphorylating MYC and constituting an AURKB/E2F1-positive feedforward loop

**DOI:** 10.1038/s41419-024-06827-y

**Published:** 2024-06-18

**Authors:** Fang Li, Xiaofei Wang, Jinyuan Zhang, Xintao Jing, Jing Zhou, Qiuyu Jiang, Li Cao, Shuang Cai, Jiyu Miao, Dongdong Tong, John Y-J. Shyy, Chen Huang

**Affiliations:** 1 Department of Cell Biology and Genetics, School of Basic Medical Sciences, Xi’an Jiaotong University School of Health Science Center, Xi’an, 710301 Shaanxi China; 2 Biomedical Experimental Center, Xi’an Jiaotong University, Xi’an, 710061 Shaanxi China; 3 Department of Hematology, The Second Affiliated Hospital of Xian Jiaotong University, Xi’an, 710004 China; 4 Division of Cardiology, Department of Medicine, University of California, San Diego, CA USA

**Keywords:** Cancer, Post-translational modifications

## Abstract

As the second most common malignant tumor in the urinary system, renal cell carcinoma (RCC) is imperative to explore its early diagnostic markers and therapeutic targets. Numerous studies have shown that AURKB promotes tumor development by phosphorylating downstream substrates. However, the functional effects and regulatory mechanisms of AURKB on clear cell renal cell carcinoma (ccRCC) progression remain largely unknown. In the current study, we identified AURKB as a novel key gene in ccRCC progression based on bioinformatics analysis. Meanwhile, we observed that AURKB was highly expressed in ccRCC tissue and cell lines and knockdown AURKB in ccRCC cells inhibit cell proliferation and migration in vitro and in vivo. Identified CDC37 as a kinase molecular chaperone for AURKB, which phenocopy AURKB in ccRCC. AURKB/CDC37 complex mediate the stabilization of MYC protein by directly phosphorylating MYC at S67 and S373 to promote ccRCC development. At the same time, we demonstrated that the AURKB/CDC37 complex activates MYC to transcribe CCND1, enhances Rb phosphorylation, and promotes E2F1 release, which in turn activates AURKB transcription and forms a positive feedforward loop in ccRCC. Collectively, our study identified AURKB as a novel marker of ccRCC, revealed a new mechanism by which the AURKB/CDC37 complex promotes ccRCC by directly phosphorylating MYC to enhance its stability, and first proposed AURKB/E2F1-positive feedforward loop, highlighting AURKB may be a promising therapeutic target for ccRCC.

## Introduction

RCC affects more than 400,000 people worldwide each year and is the second most common malignancy of the urinary system after bladder cancer [1]. According to Global Cancer Statistics 2020, the incidence of kidney cancer accounts for about 5% of all male malignancies, ranking sixth, and about 3% of female malignancies, ranking ninth [1]. According to clinicopathological characteristics, RCC can be divided into three types: ccRCC, papillary renal cell carcinoma (PRCC) and chromophobe renal cell carcinoma (chRCC), of which ccRCC accounts for about 75% and is the most common and studied subtype of RCC [2]. Since RCC is not sensitive to either chemotherapy or radiotherapy, surgery remains the effective treatment for kidney cancer that has not yet metastasized, but about 30% of patients relapse after complete removal of the primary tumor [3, 4]. Approximately 30% of patients with RCC have metastatic disease at initial presentation, and advanced RCC is a fatal disease that predicts a 5-year survival rate of only 11.7% [5, 6]. Therefore, screening the targeted molecules for early diagnosis and treatment of ccRCC and exploring the molecular mechanism of ccRCC are of great significance for the development of diagnosis and novel therapeutic strategies.

In mammals, Aurora kinases, including Aurora A, Aurora B and Aurora C, which contain a very conserved domain consisting of a short C-terminal domain, an N-terminal domain, and a protein kinase domain, plays an important role in the cell cycle [7, 8]. Aurora A and Aurora B kinase have been found to be overexpressed in a variety of cancers, acting as oncogenes, and are potential targets for cancer therapy [9, 10]. Aurora B kinase encoding gene AURKB is located in the short arm region of human chromosome 17.1 (17p13.1) and contains 344 amino acids [11]. Aurora B kinase is involved in regulating chromosome aggregation by phosphorylating Ser10 of histone H3 and Ser7 of CENP-A [12]. Studies have shown that activated AURKB mediates phosphorylation of histone H2AX at Ser121, thus promoting AURKB autophosphorylation and further accelerating AURKB activation [13]. Aurora B kinase, which is involved in cytokinesis, chromatin concentration and aggregation, chromosome segregation, spindle checkpoint regulation, and correction of misassembly of centromeres and microtubules, is upregulated in many tumor types and is associated with poor prognosis in cancer patients [14]. Aurora B kinase may have different oncogenic effects in different types of cancer. It has been shown that AURKB can regulate the G2/M transition of cell cycle by activating the expression of CCND1 and CCNB1, thereby promoting the proliferation of gastric cancer cells [15]. Previous studies have shown that AURKB is overexpressed in colon cancer, and decreased AURKB leads to activation of caspase 3, as well as decreased Mdm2, c-Myc, pAKT, and cyclin D1 [16]. Therefore, in-depth exploration of the phosphorylation substrates and upstream regulatory mechanisms of AURKB will provide important insights for the development of new diagnostic and therapeutic strategies.

Extensive genome and transcriptome sequencing highlighted that the transcription factor expression disorders mediated abnormal transcription activity of oncogene and tumor suppressor genes involved in multiple cancer diseases [17]. The E2F transcription factor family plays a key role in cell cycle regulation and apoptosis, and here we focus on E2F1 [18]. The function of E2F1 protein is regulated by the retinoblastoma protein Rb. When Rb is not in its highly phosphorylated form, it binds to E2F1 through its pocket domain and inhibits E2F1 binding to sequence-specific DNA, thereby inhibiting E2F1-mediated transcription [19, 20]. E2F1 is abnormally expressed in multiple types of cancer, including ccRCC, is directly associated with poor prognosis of cancer, and has been shown to be a key cancer biomarker [21–23].

In the current study, we used bioinformatics databases to screen AURKB as a novel ccRCC expression and prognostic marker, which was further validated by in vitro and in vivo experiments. Subsequently, identified CDC37 as a kinase molecular chaperone for AURKB, which phenocopy AURKB in ccRCC. Meanwhile, AURKB/CDC37 complex was further found to mediate the stabilization of MYC protein by directly phosphorylating MYC at S67 and S373 to promote ccRCC development. Moreover, we demonstrated that the AURKB/CDC37 complex activates MYC to transcribe CCND1, enhances Rb phosphorylation, and promotes E2F1 release, which in turn activates AURKB transcription and forms a positive feedforward loop in ccRCC. These findings will provide new insights for early diagnosis and targeted therapy of ccRCC.

## Materials and methods

### Bioinformatics data analysis

We obtained ccRCC patients’ expression data and accompanying clinical data from The Cancer Genome Atlas (TCGA) database (https://portal.gdc.cancer.gov/; https://xenabrowser.net). Three R packages DESeq2, edgeR and limma (voom) were used to screen differentially expressed genes (DEGs) in KIRC. The DEGs threshold was set at *p* value < 0.05 and |log2 fold change (FC)| > 1 to be further analyzed.

The DEGs selected by the three methods were further intersected, and the scale-free gene co-expression network was constructed using the “ weighted gene co-expression network analysis (WGCNA)” package after the qualification of DEGs was verified. Then, Pearson correlation matrices were calculated, and the weighted adjacency matrices were calculated according to the formula amn = |cmn|^β^ (cmn represents Pearson correlation between genes, amn represents adjacency between genes, and β parameter can amplify the correlation between genes). Based on the standard scale-free network, the appropriate β power was chosen. Subsequently, we transform the adjacency relationship into a topological overlap matrix (TOM) and identify modules containing similar genes by hierarchical clustering of genes. The modules of interest were identified by calculating the clinical correlation between module eigengenes (MEs) and ccRCC. After that, the log10 transform of the P-value is defined as the gene significance (GS), and the average GS of all genes in the module is defined as the module significance (MS). Finally, the module that is positively correlated with ccRCC clinical data and has the highest MS score is selected as the module related to the progress of ccRCC.

Three Machine learning methods, Gradient Boosting Machine, Random Forest and SVM-RFE, were used for binary classification feature screening of the screened red module genes to further identify the key genes.

Machine learning was further used to analyze the sensitivity of 7 key genes to ccRCC, and AUC > 0.8 was considered to have a good correlation. Subsequently, the data in Gene Expression Omnibus (GEO) database (http://www.ncbi.nlm.nih.gov/geo/) was also used for machine learning, and the genes with AUC > 0.8 in the three GEO databases were intersected to obtain 5 key genes. The raw data of GSE53757, GSE66272 and GSE53000 was obtained from the GEO database.

TCGA data were used to calculate the influence of key gene expression on prognostic data of ccRCC, including overall survival (OS), disease-specific survival (DSS) and progression-free interval (PFI). Cox regression map was used to investigate the influence of key genes on prognostic types of ccRCC. A nomogram model with important predictors was developed to predict the prognosis of ccRCC. The tumor tissues were paired with different clinical stages and pathological grades to evaluate the correlation between the expression of key genes and the clinical progression of ccRCC.

To further narrow down the candidate prognosis-related genes, the LASSO Cox regression prognosis model was constructed according to the formula riskScore = geneExp*Coef.

All statistical analyses were performed with R software v 4.2.2. R language description was shown in Table S1.

### Immunohistochemical (IHC)

All ccRCC tissues and counterparts’ tissues were obtained from Shaanxi Provincial People’s Hospital. No local or systemic treatment had been conducted before the operation. Informed consent was obtained from each patient, and the study was approved by the Biomedical Ethics Committee of Medical Department of Xi’an Jiaotong University. The IHC was performed using the human Biotin-Streptavidin HRP Detection kit (cat. no. SP-9001; ZSGB-BIO) according to the previous description. The primary antibody was anti-AURKB (1:300 dilution).

### Cell culture

786-O and HK-2 cells were maintained in RPMI‑1640 medium (Gibco-BRL, NY, USA) supplemented with 10% fetal bovine serum (FBS) and 1% penicillin/streptomycin. CAKI-1 cells were maintained in McCoy’s 5a Modified Medium (Gibco-BRL, NY, USA) supplemented with 15% FBS and 1% penicillin/streptomycin. 293T and HEK-293 cells were maintained in DMEM medium (Gibco-BRL, NY, USA) supplemented with 10% FBS and 1% penicillin/streptomycin. Cells were maintained in a 37°C, 5% CO_2_ incubator. All cell lines were sourced from the Key Laboratory of Environment and Genes Related to Diseases at Xi’an Jiaotong University (Xi’an, China).

### Small interfering RNA (siRNA) synthesis, plasmid construction, and transfection

siRNAs and the negative control (siNC) were synthesized by GenePharma (Shanghai, China) and the specific siRNA sequences are listed in Table S2. The plasmids (Wild type, WT) were purchased from Genechem (Shanghai, China). The mutant type (MUT) plasmids (Flag-AURKB K106R, His-MYC S67D, His-MYC S67A, His-MYC S373D, His-MYC S373A, His-MYC S67D S373D and His-MYC S67A S373A) were constructed using the Fast Mutagenesis System kit (TransGen Biotech) based on the WT plasmids following the manufacturer’s method. Using jetPRIME™ reagent (Polyplus-Transfection, France) for siRNA and siNC transfection according to the manufacturer’s method.

### RNA isolated, reverse transcription, and quantitative real-time PCR (qRT-PCR)

As previously described [24], total RNA was isolated using the TRIzol reagent (Genestar, Beijing, China). Reverse transcription was conducted using Hifair® II 1st Strand cDNA Synthesis SuperMix for qPCR (Yeasen, Shanghai, China) to synthesize cDNAs to mRNAs according to the manufacturer’s method. qRT-PCR was performed using Hieff® qPCR SYBR Green Master Mix (No Rox) (Yeasen, Shanghai, China) on the iQ5 Multicolor qRT-PCR system (Bio-Rad, USA). The relative expression of the experimental groups was calculated by the comparative 2^−ΔΔCt^ method and GAPDH was used as an internal control. All primers used in the present study are listed in Table S2.

### Immunoprecipitation and western blotting

Proteins were extracted from cells and tissues using a modified buffer (RIPA/Mammalian Cell Lysis Reagent, protease inhibitor and phosphatase inhibitor), followed by immunoprecipitation and western blotting with the corresponding antibodies. For immunoprecipitation, cell lysates removed from cell debris were incubated with the specified antibody at 4 °C overnight, followed by Dynabeads (Invitrogen, USA) for 4 h, and the beads were boiled after extensive washing. An equivalent protein sample was isolated by SDS-PAGE, transferred to a PVDF membrane (Millibo), and incubated with the specified antibody for detection. All primary and secondary antibodies used in the present study are listed in Table S2.

### MTT assay

Cells (3 × 10^3^) were seeded into 96-well plates and transfection was carried out with siRNAs or plasmids the next day. 10 μl MTT (5 mg/mL) was added at 24/48/72 h, discarded the supernatant and added 150 μl dimethyl sulfoxide (DMSO) after incubated at 37 °C for 4 h. The absorbance of the sample at 492 nm was measured using a microplate reader (FLUO star OPTIMA, BMG, Germany).

### Colony forming assay

Cells were trypsinized and seeded in 6/12-well plates (500/1000 cells/well). After cultured for 10–14 days, cells were fixed with 4% paraformaldehyde and stained with 0.1%crystal violet. Colony images were acquired and colony numbers were analyzed using Quantity One Software (Bio-Rad, USA).

### Cell apoptosis assay

The cells were trypsinized and washed twice with PBS. Subsequently, the cells were stained using Annexin-V-FITC/PI apoptosis detection kit (Yeasen, Shanghai, China) according to the manufacturer’s method. Cell apoptosis was analyzed using flow cytometry (FACSCalibur, BD, USA).

### Cell cycle assay

The cells were trypsinized and washed twice with PBS, and fixed in ice‑cold 75% alcohol at 4 °C overnight. After washed twice with PBS, the cells were suspended with equal volumes of RNase A (0.1 mg/ml) and propidium iodide (PI, 0.05 mg/ml) for 30 min at room temperature. Cell cycle distributions were measured by flow cytometry (FACSCalibur, BD, USA).

### Cell migration assay

Wound healing assay and transwell assay were used to detect the cells migration ability in vitro.

For wound healing assay, use a 200 μl pipette tip to make a wound after 4–6 h of transfection. Take pictures at 0 h, 12 h, 24 h, 36 h and 48 h to detect wound closure and relative migration rates were quantified using image J (Wayne Rasband, USA).

For transwell assay, 200 μl FBS-free medium containing 2 × 10^4^ cells were added to the upper chamber (Millipore, Billerica, MA, USA) and 600 μl complete medium was added to the bottom of the chamber. After continued incubation of cells for 24 h/48 h, cells left on the upper surface were removed, whereas cells that migrate to the bottom surface were fixed in 4% polyformaldehyde and stained with 0.1% crystal violet. After acquiring images of cells migrating to the bottom surface using a microscope at 10 × magnification, the migrated cells were dissolved in 33% glacial acetic acid, and the absorbance of the sample was measured at 570 nm using a microplate reader (FLUO star OPTIMA, BMG, Germany).

### Animal experiment

The shAURKB lentivirus vector was purchased from Genechem (Shanghai, China) and the sequence was consistent with siAURKB-2. We generated CAKI-1 cells that stably silenced AURKB for Animal experiment. The 5-week-old male BALB/C-nude mice were purchased from the Experimental Animal Center of Xi’an Jiaotong University. All animal experiments were approved by the Medical Ethics Committee of Xi’an Jiaotong University (No. 2023-2240) and were performed according to the institution’s guidelines for the use of laboratory animals.

For xenograft assay, 1 × 10^6^ LV-control and LV-shAURKB cells were subcutaneously injected into both sides of the groin of nude mice (*n* = 4/group). Tumor size was measured using vernier calipers every 3 days and tumor volume was calculated according to the formula: *V* = (*L* × *W*^2^)/2. After 28 days of injection, nude mice intraperitoneally injected with d-Luciferin potassium salt (Beyotime, Shanghai, China) were placed under isoflurane/oxygen anesthesia in the IVIS Spectrum (Xenogen, Alameda, CA, USA) and bioluminescence imaging in vivo was obtained. Then the nude mice were sacrificed and xenograft tumors were surgically removed. After the tumor was weighed and photographed, the tissue was frozen for qRT-PCR and Western blotting.

For tumor metastasis assay, 1 × 10^6^ LV-control and LV-shAURKB cells were injected into nude mice by tail vein (*n* = 4/group). After 21 days of injection, nude mice intraperitoneally injected with d-Luciferin potassium salt (Beyotime, Shanghai, China) were placed under isoflurane/oxygen anesthesia in the IVIS Spectrum (Xenogen, Alameda, CA, USA)) and bioluminescence imaging in vivo was obtained. Then the nude mice were sacrificed, the organs were surgically removed and the images of the metastases were taken with IVIS Spectrum (Xenogen, Alameda, CA, USA).

### Cell immunofluorescence assay

In brief, cells were fixed with 4% paraformaldehyde for 15 min, permeabilized with PBS-T (0.2% Triton X-100) for 20 min, followed by blocking using PBS-B (4% BSA) for 1 h at room temperature. Cells were then incubated with primary antibodies (His/Flag) at 4 °C overnight, and stained using appropriate fluorochrome-conjugated secondary antibody for 1 h at room temperature in the dark. After staining the nucleus with 4’6-diamidino-2-phenylindole (DAPI) (0.1 mg/ml) (Beyotime, Beijing, China), immunofluorescence signals were detected by fluorescence microscopy (Leica, TCS SP8 DIVE, Germany).

### Chromatin Immunoprecipitation (ChIP)

Cells were cross-linked with 1% formaldehyde at room temperature for 15 min and quenched with 125 mM glycine. The cells were sonicated to break the chromatin into fragments of approximately 200 bp. Primary antibodies were added to the lysate and incubated at 4 °C overnight. Dynabeads (Invitrogen, USA) was subsequently added to capture the DNA-protein complex. Added sodium chloride to reverse crosslinking at 65 °C for 7 h, and DNA was extracted using phenol chloroform‒isoamyl alcohol. After that, qRT-PCR and agarose gel electrophoresis were performed for the analysis of ChIP products.

### Dual-luciferase reporter assay

HEK-293 cells were seeded in 96‐well plates. The next day, a pGL3 promoter luciferase vector containing the AURKB/CCND1 promoter region was co‐transfected with the indicated plasmids or siRNAs, respectively. Luciferase activity was measured on a microplate reader (FLUO star OPTIMA, BMG, Germany) using the dual luciferase assay system (Promega) according to the manufacturer’s method after 48 h. The activity of renal luciferase was used as the internal standard.

### Statistical analysis

Statistical analysis was performed using SPSS Statistics 18.0 (Chicago, IL, USA) and results were shown as mean ± standard deviation (SD) of at least three different experiments. Student t test was used for comparison between two independent groups. Differences between more than two groups were analyzed by one-way ANOVA followed by Dunnett’s test for multiple comparisons. p < 0.05 was considered statistically significant.

## Results

### Bioinformatics analysis showed that oncogene AURKB may be a new diagnostic biomarker and prognostic biomarker in ccRCC

**Fig. 1:**
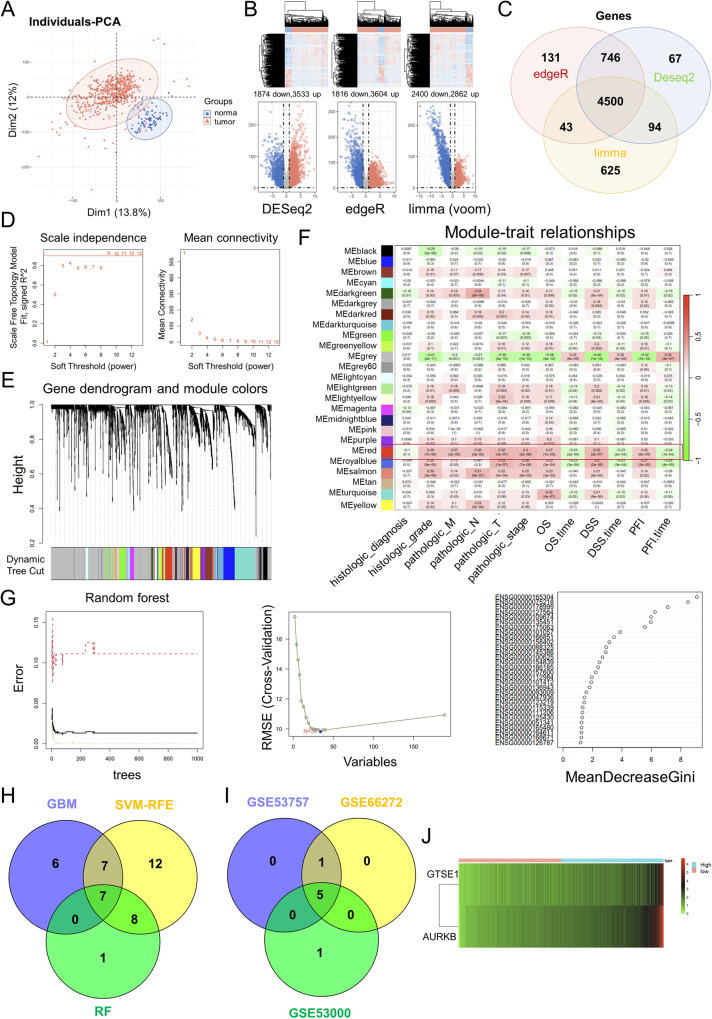
Bioinformatics databases identified AURKB as a novel prognostic key gene for ccRCC. **A** PCA of ccRCC-related genes in TCGA database. **B** Three R packages DESeq2, edgeR and limma (voom) were used to screen for differentially expressed genes (DEGs) in ccRCC and genes with p value < 0.05 and log2 fold change (FC)| > 1 were DEGs. **C** Venn diagram shows the intersection of DEGs screened for ccRCC using three R packages DESeq2, edgeR, and limma (voom), with a total of 4500 DEGs. **D** Soft-thresholding powers selection. **E** WGCNA cluster dendrogram and module assignment. **F** Scale-free gene co-expression network was constructed using the “WGCNA” package, and the red module was identified as the module with the strongest correlation with clinical stage and survival. **G** Three Machine learning methods, Gradient Boosting Machine, Random Forest and SVM-RFE, were used for binary classification feature screening of the screened red module genes. **H** Venn diagram shows the intersection of seven genes screened by three machine learning methods, gradient boosting machine, random forest and SVM-RFE. **I** The Venn diagram shows the intersection of validation results for three GEO databases. **J** LASSO Cox regression prognosis model was constructed according to the formula riskScore = geneExp*Coef.

In the current study, we hope to utilize ccRCC data from bioinformatics databases to screen for new ccRCC diagnostic and prognostic biomarkers. We first performed principal component analysis (PCA) on the ccRCC-related genes in TCGA database (Fig. 1A). Three R packages DESeq2, edgeR and limma (voom) were used to screen for DEGs in ccRCC and genes with p value < 0.05 and log2 fold change (FC)| > 1 were DEGs (Fig. 1B). The DEGs screened by the three methods were further crossed and a total of 4500 DEGs were obtained (Fig. 1C).

The scale-free gene co-expression networks were constructed using the “WGCNA” package after the qualification of DEGs was verified. When we set the soft-threshold power β to 9, the links between genes in the gene network conform to the scale-free network distribution (Fig. 1D). A total of 25 co-expressed modules were identified (Fig. 1E). We subsequently came to verify the correlation of each module with ccRCC clinical stage and survival, and the red module, which showed higher MS values and greater correlation with clinical stage and survival, was identified as the most relevant module (Fig. 1F).

Three Machine learning methods, Gradient Boosting Machine, Random Forest and SVM-RFE, were used for binary categorical feature screening of the screened red module genes, further identified the seven hub genes (AURKB, GTSE1, MELK, SIX4, TMEM164, TROAP and UBE2C) (Fig. 1G, H).

Machine learning was further used to analyze the sensitivity of 7 key genes to ccRCC, with AUC > 0.8 considered a good correlation (Fig. S1A). Subsequently, the data in GEO database was also used for machine learning (Fig. S1B–D), and the genes with AUC > 0.8 in the three GEO databases were intersected to obtain 5 hub genes (AURKB, GTSE1, MELK, SIX4 and TMEM164) (Fig. 1I).

Moreover, AURKB, GTSE1, MELK and SIX4 were found to be negatively correlated with OS, DSS and PFI (Fig. S2A). The same results were obtained in cox regression map (Fig. S2B). AURKB, GTSE1 and MELK were significantly up-regulated in ccRCC, and their high expression were positively correlated with the TNM stage of ccRCC (Fig. S3A–D).

To further narrow down the candidate prognosis-related genes, the LASSO Cox regression prognosis model was constructed according to the formula riskScore = geneExp*Coef (Fig. 1J), and the correlation coefficient for AURKB was greater (GTSE1 coef = 0.016, AURKB coef=0.021), suggesting a greater role for AURKB in ccRCC prognosis. The above analysis suggests that the oncogene AURKB may be a new diagnostic biomarker and prognostic biomarker for ccRCC.

### AURKB is up-regulated in ccRCC and promotes the proliferation and migration of ccRCC cells in vitro in vivo

**Fig. 2:**
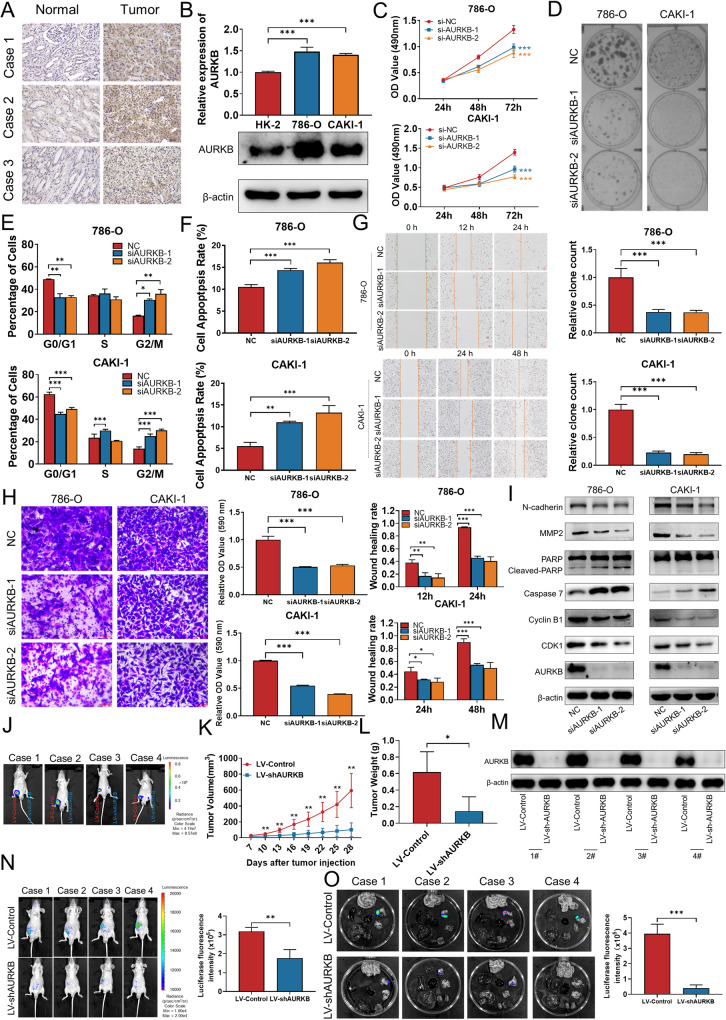
AURKB is up-regulated in ccRCC and promotes the proliferation and migration of ccRCC cells in vitro in vivo. **A** Representative immunohistochemical images of AURKB in human ccRCC tissues and adjacent normal tissues. **B** qRT-PCR and western blotting were used to detect the mRNA and protein expression of AURKB. **C** MTT assay was used to detect the effect of AURKB knockdown on the proliferation of ccRCC cells. **D** Colony formation assay was used to detect the effect of AURKB knockdown on the colony formation ability of ccRCC cells. **E** The effect of AURKB knockdown on cell cycle of ccRCC cells was detected by flow cytometry. **F** Flow cytometry was used to detect the effect of AURKB knockdown on cell apoptosis of ccRCC cells. **G** Wound healing assay was used to detect the effect of AURKB knockdown on the migration of ccRCC cells. **H** Transwell assay was used to detect the effect of AURKB knockdown on the migration of ccRCC cells, and the OD value of the traversed cells was used for statistical mapping. **I** Western blotting was used to detect the effect of AURKB knockdown on the expression of cell cycle, apoptosis and migration related molecules at protein level in 786-O and CAKI-1. **J** On the day 28, tumor was measured by in vivo bioluminescence imaging. **K** Tumor growth curves of the tumor volumes represent measurements taken every 3 d for 28 d. **L** The xenograft tumors were weighed and statistically mapped. **M** The protein levels of AURKB in xenograft tumors were analyzed by western blotting. **N** Image analysis was performed on nude mice to assess tumor metastasis on the 21th day after injection. **O** Image analysis was performed on the isolated organs of nude mice on day 21 after injection to assess tumor metastasis. *p < 0.05, **p < 0.01, ***p < 0.001.

Consistent with the bioinformatics analysis, the IHC results showed that AURKB expression was significantly higher in ccRCC tissues than that in adjacent normal tissues (Fig. 2A). Next, we analyzed AURKB expression in two ccRCC cell lines 786-O, CAKI-1, and normal renal epithelial cell line HK-2. qRT-PCR and western blotting results showed that AURKB mRNA and protein levels were up-regulated in 786-O and CAKI-1 compared with HK-2 (Fig. 2B).

To further explore the biological function of AURKB in ccRCC cells, we designed and synthesized two siRNAs targeting AURKB. The knockdown efficiency of siRNA was detected by qRT-PCR, and the expression of AURKB was effectively knocked down as shown in Fig. S4A. Cell proliferation was inhibited after knockdown AURKB in 786-O and CAKI-1 cells according to MTT assay (Fig. 2C) and colony formation assay (Fig. 2D). In order to investigated whether AURKB knockdown inhibits ccRCC cell proliferation by regulating apoptosis and cell cycle, we performed cell cycle analysis and cell apoptosis analysis using flow cytometry. The percentage of siAURKB transfected 786-O and CAKI-1 cells in the G2 phase increased, while the percentage of cells in G1 phase decreased compared with siNC and this suggests that knockdown of AURKB causes ccRCC cell cycle arrest at G2 phase (Fig. 2E and Fig. S4B). Meanwhile, flow cytometry results indicated that knockdown of AURKB was found to significantly induce the ccRCC cell apoptosis (Fig. 2F and Fig. S4C). Subsequently, wound healing and transwell assays were performed to clarify the effect of AURKB knockdown on ccRCC cell migration ability. As shown in Fig. 2G, knockdown of AURKB significantly inhibited wound healing rate in ccRCC cells. Consistent with the wound healing results, the migration ability of cells in the AURKB knockdown group was significantly inhibited compared with the control group in the transwell assay (Fig. 2H). Furthermore, western blotting analysis was performed to investigate the molecular mechanism of AURKB regulating the biological functions of ccRCC. Western blotting results showed that after AURKB knockdown in ccRCC cells, cell cycle G2-related proteins CDK1 and Cyclin B1 were decreased, pro-apoptotic proteins Caspase 7 and cleaved PARP were increased and migration promoting proteins N-cadherin and MMP2 were down-regulated (Fig. 2I).

To demonstrate the carcinogenic effect of AURKB in vivo, we generated CAKI-1 cells that stably knockdown AURKB to perform animal experiments (Fig. S4D). For xenograft assay, LV-Control and LV-shAURKB transfected CAKI-1 cells were subcutaneously injected into the bilateral groin of BALB/C nude mice. On the 28th day, the tumor formed on the side of LV-shAURKB in the same nude mouse was significantly smaller than LV-Control group (Fig. 2J and Fig. S4E, F). The tumor growth curve was plotted using the tumor volume measured during tumor formation, and the results showed that tumors in the LV-shAURKB group grew slower than those in the LV-Control group (Fig. 2K). Knockdown of AURKB significantly reduced tumor volume and tumor weight compared with LV- Control (Fig. 2L). Expression of AURKB in tumor tissues was detected by qRT-PCR and western blotting. The results demonstrated that AURKB expression was significantly lower in LV-shAURKB infected tumors than LV-Control (Fig. 2M and Fig. S4G). For tumor metastasis assay, LV-Control and LV-shAURKB transfected CAKI-1 cells were injected into nude mice by tail vein and detected the distribution and size of the tumor on the 21th day after injection. Compared with the LV-Control group, the LV-shAURKB group showed less tumor metastasis (Fig. 2N, O). Taken together, these results indicated that AURKB is up-regulated in ccRCC and promotes the proliferation and migration of ccRCC cells in vitro in vivo.

### Identification of CDC37 as a molecular chaperone for AURKB and CDC37 phenocopy AURKB in ccRCC

**Fig. 3:**
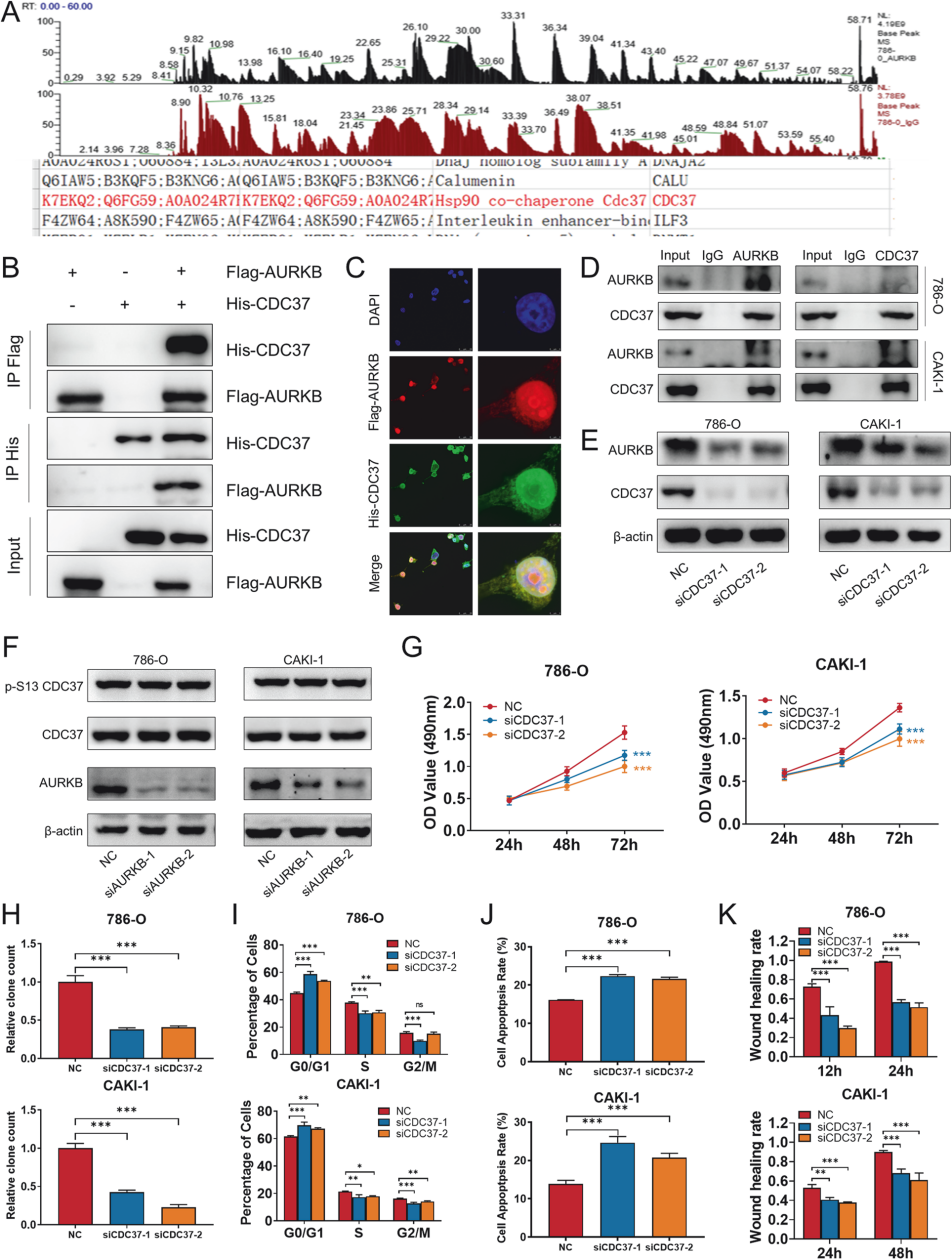
Identification of CDC37 as a AURKB binding partner and CDC37 phenocopy AURKB in ccRCC. **A** Co-IP mass spectrometry identified CDC37 as the binding partner of AURKB. **B** Lysates of 293T cells overexpressing Flag-AURKB and/or His-CDC37 were subjected to reciprocal Co-IP to detect protein interaction. **C** Representative images of immunofluorescence staining of DAPI, Flag-AURKB and His-CDC37 in 293T cells. **D** 786-O and CAKI-1 cell lysates were subjected to Co-IP to detect endogenous CDC37 and AURKB interaction. **E** Western blotting was used to detect the expression changes of target proteins after CDC37 knockdown in 786-O and CAKI-1 cells, with β-actin as a control. **F** Western blotting was used to detect the expression changes of target proteins after AURKB knockdown in 786-O and CAKI-1 cells, with β-actin as a control. **G** MTT assay was used to detect the effect of CDC37 knockdown on the proliferation of 786-0 and CAKI-1 cells. **H** Colony formation assay was used to detect the effect of CDC37 knockdown on the colony formation ability of 786-0 and CAKI-1 cells. **I** The effect of CDC37 knockdown on cell cycle of 786-O and CAKI-1 cells was detected by flow cytometry, and the percentage of cells in each period was plotted. **J** Flow cytometry was used to detect the effect of CDC37 knockdown on cell apoptosis of 786-O and CAKI-1 cells, and the percentage of apoptotic cells was plotted. **K** Wound healing assay was used to detect the effect of CDC37 knockdown on the migration of 786-O and CAKI-1 cells, and the percentage of wound healing was plotted. *p < 0.05, **p < 0.01, ***p < 0.001.

The AURKB binding immunoprecipitates in 786-O cells were analyzed by liquid chromatography-tandem mass spectrometry, which led to the identification of CDC37 as a AURKB binding partner (Fig. 3A). To validate the physical interaction, we enforced the expression of Flag-AURKB and His-CDC37 in 293T cells for reciprocal immunoprecipitation and immunofluorescence and confirmed associations between both proteins (Fig. 3B, C). Meanwhile, co-immunoprecipitation (Co-IP) using 786-O and CAKI-1 cell lysates validated the specific interaction between endogenous AURKB and CDC37 in these two cells (Fig. 3D). Interestingly, knockdown of CDC37 reduced AURKB expression (Fig. 3E) while AURKB knockdown had no effect on CDC37 protein expression and phosphorylation in 786-O and CAKI-1 cells (Fig. 3F). Therefore, we propose the hypothesis that CDC37 is involved in the phosphorylation of AURKB downstream molecules as a molecular chaperone of AURKB, and then we will conduct the effect of CDC37 on AURKB downstream to verify this conjecture.

To investigate the biological function of CDC37 in ccRCC cells, we synthesized two small interfering RNAs targeting CDC37 and determined that their knockdown efficiency was high (Fig. S5A). MTT assay was performed to determine the viability of ccRCC cells, and it was found that knockdown of CDC37 significantly inhibited cell proliferation (Fig. 3G). Consistent with the MTT results, the results of the clone formation assay also showed that inhibition of CDC37 expression reduced the number and size of colonies formed (Fig. 3H and Fig. S5B). Based on the results of cell cycle and apoptosis studies by flow cytometry, knockdown of CDC37 induced G1 phase cell cycle arrest, while reducing the percentage of S phase cells (Fig. 3I and Fig. S5C) and promoting apoptosis (Fig. 3J and Fig. S5D). In addition, both wound healing and permeability assays showed that knockdown of CDC37 significantly inhibited the migratory ability of ccRCC cells compared with the control group (Fig. 3K and Fig. S5E, F). Furthermore, western blotting analysis was performed to investigate the molecular mechanism by which CDC37 regulates the biological functions of ccRCC. Western blotting results showed that after CDC37 knockdown in ccRCC cells, cell cycle G1-related proteins CDK4 and Cyclin D1 were decreased, pro-apoptotic protein Bax increased while Bcl-2 decreased, and migration promoting protein MMP2 was down-regulated (Fig. S5G). These results suggest that CDC37 phenocopy AURKB in ccRCC.

### AURKB-mediated MYC phosphorylation contributes to MYC stability

**Fig. 4:**
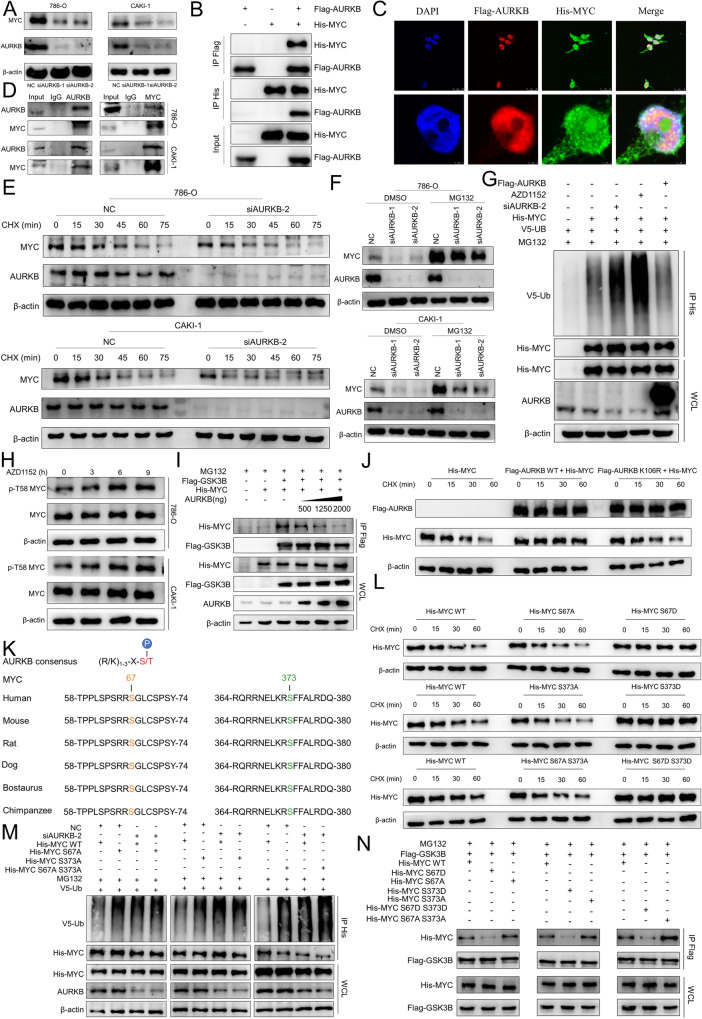
AURKB-Mediated MYC Phosphorylation Contributes to MYC Stability. **A** Western blotting was used to detect the expression changes of MYC after AURKB knockdown in ccRCC cells. **B** Lysates of 293T cells overexpressing Flag-AURKB and/or His-MYC were subjected to reciprocal Co-IP to detect protein interaction. **C** Representative images of immunofluorescence staining of DAPI, Flag-AURKB and His-CDC37 in 293 T cells. **D** 786-O and CAKI-1 cell lysates were subjected to Co-IP to detect endogenous MYC and AURKB interaction. **E** Time course analysis of MYC protein level in AURKB knockdown ccRCC cells. **F** AURKB knockdown ccRCC cells were treated with MG132 (10 μM) for 6 h before harvest. AURKB and MYC were analyzed by immunoblot. **G** HEK-293T cells were co-transfected and treated with MG132 (10 μM) for 6 h before harvest. Cell lysates were subjected to Co-IP, ubiquitination, and immunoblot assays. **H** Time course analysis of MYC and p-T58 MYC by immunoblot upon AZD1152 (786-O: 500 nM; CAKI-1: 300 nM) treatment in ccRCC cells. **I** HEK-293T cells were co-transfected with Flag-GSK3β, His-MYC, and/or increasing doses of AURKB (0.5,1.25, and 2 μg) and treated with MG132 (10 μM) for 6 h before harvest. Cell lysates were subjected to Co-IP and immunoblot assays. **J** Time-course analysis of His-MYC levels was performed in 293T cells expressing ectopic His-MYC, Flag-AURKB WT or a mutant (K106R). **K** Peptide sequence alignment of MYC in different species. **L** Time-course analysis of His-tagged MYC by immunoblot in 293T cells. **M** HEK-293T cells were co-transfected and treated with MG132 (10 μM) for 6 h before harvest. Cell lysates were subjected to Co-IP, ubiquitination, and immunoblot assays. **N** HEK-293T cells were co-transfected and treated with MG132 (10 μM) for 6 h before harvest. Cell lysates were subjected to Co-IP and immunoblot assays.

We next searched for phosphorylated substrates of AURKB to explore the molecular mechanism by which it promotes ccRCC. Knockdown of AURKB or AZD1152 reduced the steady-state levels of MYC protein in 786-O and CAKI-1 cells (Fig. 4A and Fig. S6A). Next, we explore whether AURKB and MYC have physical interaction. We enforced the expression of Flag-AURKB and His-MYC in 293T cells for reciprocal immunoprecipitation and immunofluorescence and confirmed associations between both proteins (Fig. 4B, C). Meanwhile, Co-IP using 786-O and CAKI-1 cell lysates validated the specific interaction between endogenous AURKB and MYC in these two cells (Fig. 4D). Actually, time-course experiments showed that knockdown of AURKB significantly reduced the half-life of endogenous MYC in 786-O and CAKI-1 cells (Fig. 4E and Fig. S6B). Meanwhile, MG132 effectively rescued the decreasing effect of AURKB knockdown on MYC protein abundance, suggesting that knockdown of AURKB stimulates proteasomal degradation of MYC (Fig. 4F). The same results were obtained for AZD1152 treatment (Fig. S6C-E).

Regulation of MYC degradation by the ubiquitin-proteasome system is dependent on T58 phosphorylation of MYC mediated by GSK3β [25]. Co-IP analysis showed that AURKB knockdown directly increased the ubiquitination of exogenous MYC, and the results were consistent when HEK-293T cells were treated with the AURKB inhibitor AZD1152, and conversely the increased AURKB expression decreased the ubiquitination of exogenous MYC (Fig. 4G). In addition, we performed time-course analysis to examine whether AURKB inhibition affected MYC p-T58 and showed that AZD1152 treatment resulted in a significant increase in MYC p-T58 in 786-O and CAKI-1 cells, suggesting inactivation of AURKB enhances GSK3β-mediated T58 phosphorylation (Fig. 4H). Conversely, increasing doses of AURKB expression abolished MYC and GSK3β interaction (Fig. 4I). AURKB expression significantly prolonged the half-life of exogenous MYC, while AURKB deactivation mutant kinase (K106R) failed to prolong its half-life, suggesting that the stability of MYC need AURKB kinase activity (Fig. 4J and Fig. S6F).

Next, we explored whether AURKB could directly phosphorylate MYC. We found that MYC protein sequences contain potential sites that match the AURKB phosphorylation consensus motif. The potential phosphorylated residues serine 67 (S67), phosphorylated residue serine 373 (S373) and their surrounding amino acids are highly conserved in a variety of mammals (Fig. 4K). We subsequently generated MYC non-phosphorylatable alanine mutants (S67A, S373A, S67A S373A) and phosphomimetic aspartic acid mutants (S67D, S373D, S67D S373D) using site-directed mutagenesis kit, respectively, and performed a series of cycloheximide chase experiments to assess the half-life of these mutant proteins. Compared with the wild type, the S67D, S373D and S67D S373D mutations increased the half-life of MYC, and the S67A, S373A and S67A S373A mutations further reduced the half-life of MYC (Fig. 4L and Fig. S6G).

To further demonstrate that MYC S67 and S373 phosphorylation inhibited ubiquitination, we added siAURKB or siNC to HEK-293T cells overexpressing MYC WT or S67A, S373A, and S67A S373A mutants, respectively. The results showed the treatment with siAURKB led to MYC WT ubiquitination levels increased dramatically, however there were no significant changes in S67A, S373A, and S67A S373A three mutants (Fig. 4M). Consistent results could be observed in HEK-293T cells with AZD1152 added (Fig. S6H). Consistent with these results, the S67D, S373D and S67D S373D mutants diminished, whereas the S67A, S373A and S67A S373A mutants enhanced the binding with GSK3β (Fig. 4N).

### CDC37 acts as a molecular chaperone for AURKB to enhance MYC stability

**Fig. 5:**
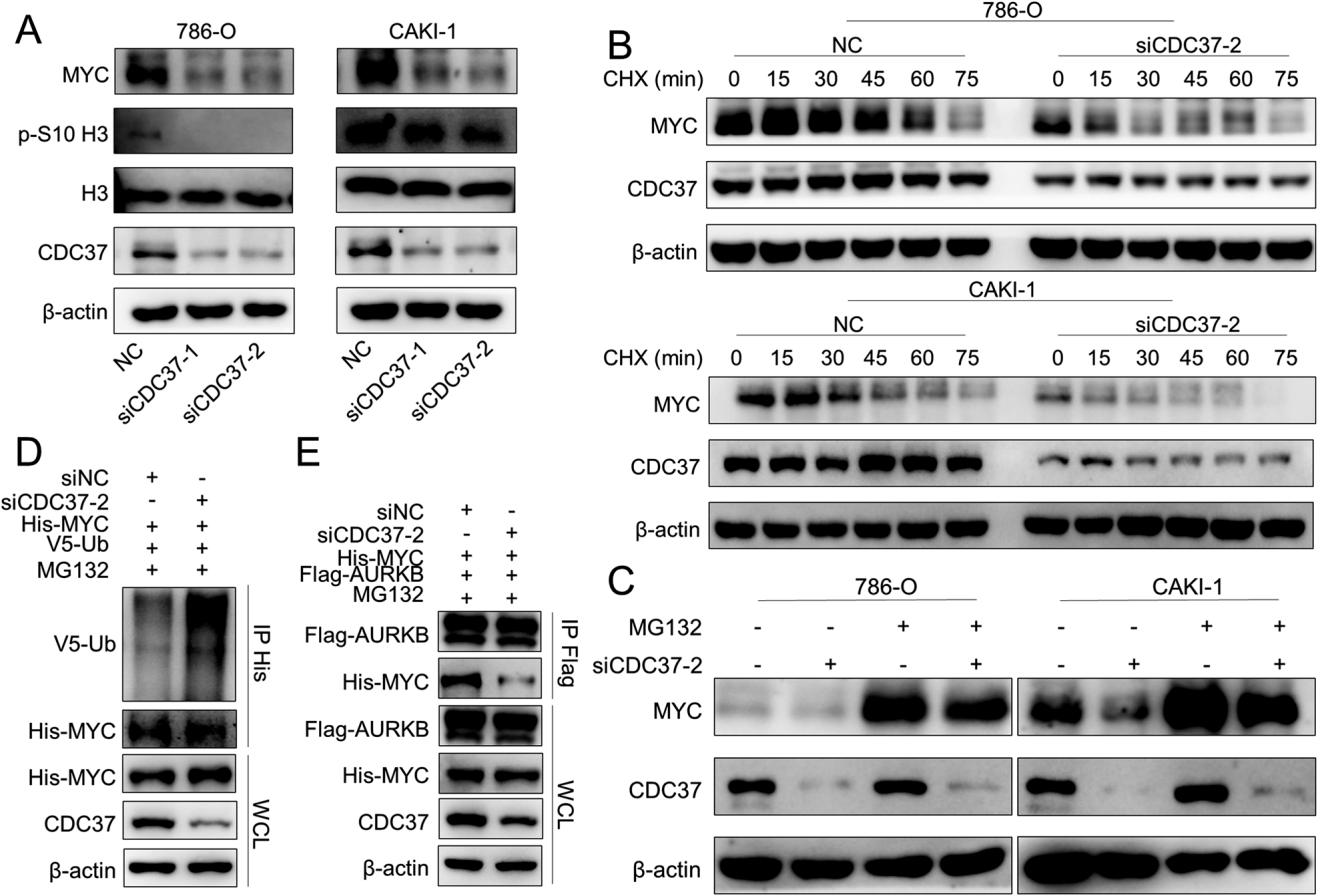
CDC37-mediated binding of AURKB to MYC contributes to MYC Stability. **A** Western blotting was used to detect the expression changes of target proteins after CDC37 knockdown in 786-O and CAKI-1 cells, with β-actin as a control. **B** Time course analysis of MYC protein levels in CDC37 knockdown 786-O and CAKI-1 cells. **C** CDC37 knockdown 786-O and CAKI-1 cells were treated with MG132 (10 μM) for 6 h before harvest. CDC37 and MYC were analyzed by immunoblot, with β-actin as a control. **D** HEK-293T cells were co-transfected with NC+His-MYC + V5-UB or siCDC37+His-MYC + V5-UB and treated with MG132 (10 μM) for 6 h before harvest. Cell lysates were subjected to Co-IP, ubiquitination, and immunoblot assays. **E** HEK-293T cells were co-transfected with NC+His-MYC+Flag-AURKB or siCDC37+His-MYC+Flag-AURKB. Cell lysates were subjected to Co-IP and immunoblot assays.

Previously, we have confirmed that CDC37 is a binding partner of AURKB and proposed the hypothesis that CDC37 is involved in the phosphorylation of AURKB downstream molecules as a molecular chaperone of AURKB. Previous studies have shown that AURKB mediates serine 10 phosphorylation of histone H3 [12], and we demonstrated in the previous section that AURKB-mediated MYC phosphorylation contributes to MYC stability. Therefore, we confirmed our hypothesis by examining whether CDC37 affects the phosphorylation of histone H3 serine 10, the rate of ubiquitin degradation of MYC, and the binding of AURKB to MYC. First of all, we examined the expression of histone H3, serine 10 phosphorylated histone H3 and MYC in AURKB knockdown 786-O and CAKI-1 cells. CDC37 depletion reduced histone H3 phosphorylation of the serine 10 and the steady-state level of MYC protein (Fig. 5A). Actually, time-course experiments showed that knockdown of CDC37 significantly reduced the half-life of endogenous MYC in 786-O and CAKI-1 cells (Fig. 5B and Fig. S7A, B). Meanwhile, MG132 effectively rescued the decreasing effect of CDC37 knockdown on MYC protein abundance, suggesting that knockdown of CDC37 stimulates proteasomal degradation of MYC (Fig. 5C). Further, co-immunoprecipitation (Co-IP) analysis showed that CDC37 knockdown directly increased MYC ubiquitination (Fig. 5D). In addition, CDC37 depletion eliminates MYC and AURKB interactions (Fig. 5E). These results suggest that CDC37 may maintain the stability of MYC by mediating AURKB and MYC interaction to reduce MYC ubiquitination.

### Enhanced MYC expression rescued the effects of AURKB/CDC37 depletion on ccRCC cells

**Fig. 6:**
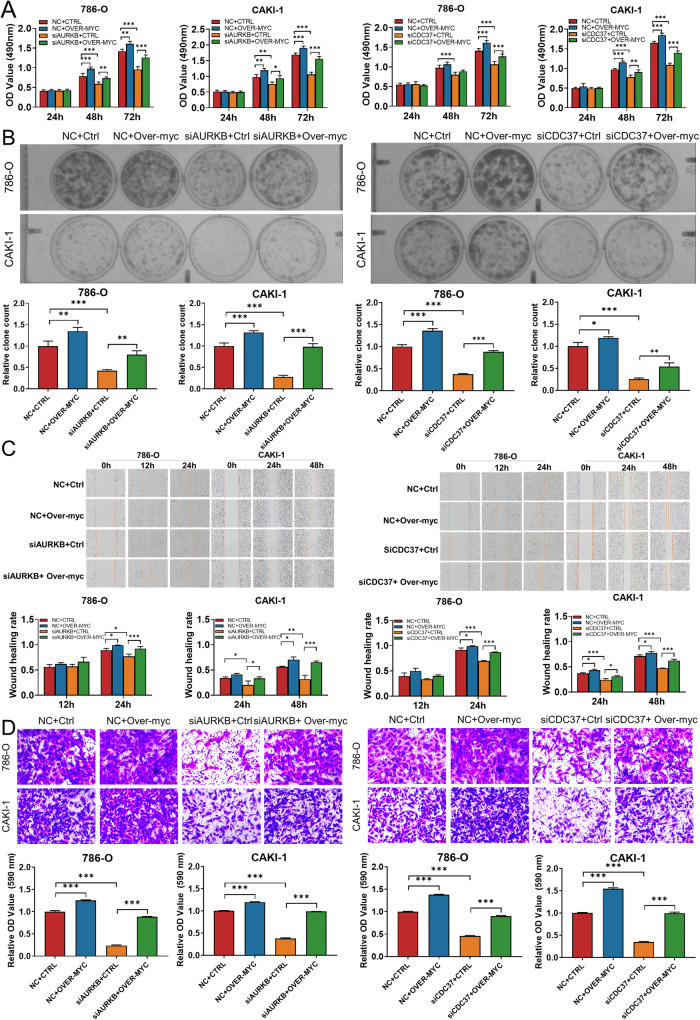
Enhanced MYC expression rescued the effects of AURKB/CDC37 depletion on ccRCC cells. **A**, **B** MTT and colony formation assays were performed to determine the impact of cell viability treated with NC+Ctrl, NC+Over-MYC, siAURKB-2+Ctrl, siAURKB-2+Over-MYC, siCDC37-2+Ctrl, siCDC37-2+ Over-MYC in ccRCC cells. **C**, **D** Transwell and wound‐healing analysis represented the migration and metastasis capacity of ccRCC cells co‐transfected with NC+Ctrl, NC+Over-MYC, siAURKB-2+Ctrl, siAURKB-2+ Over-MYC, siCDC37-2+Ctrl, siCDC37-2+ Over-MYC. *p < 0.05, **p < 0.01, ***p < 0.001.

Since depletion of AURKB/CDC37 both accelerates the degradation of MYC, we wondered whether enhanced MYC expression could rescue the inhibitory effects of AURKB/CDC37 depletion on the proliferation and migration of ccRCC cells. The results of MTT (Fig. 6A), colony forming (Fig. 6B), wound healing (Fig. 6C) and transwell (Fig. 6D) assays demonstrated that enhanced MYC expression partially reversed the inhibitory effects of AURKB/CDC37 depletion on ccRCC cell proliferation and migration. This suggests that AURKB/CDC37 complexes promote ccRCC cell proliferation and migration by phosphorylating and stabilizing MYC.

### AURKB/CDC37 regulates MYC transcriptional program and promotes the phosphorylation of Rb1 to release E2F1

**Fig. 7:**
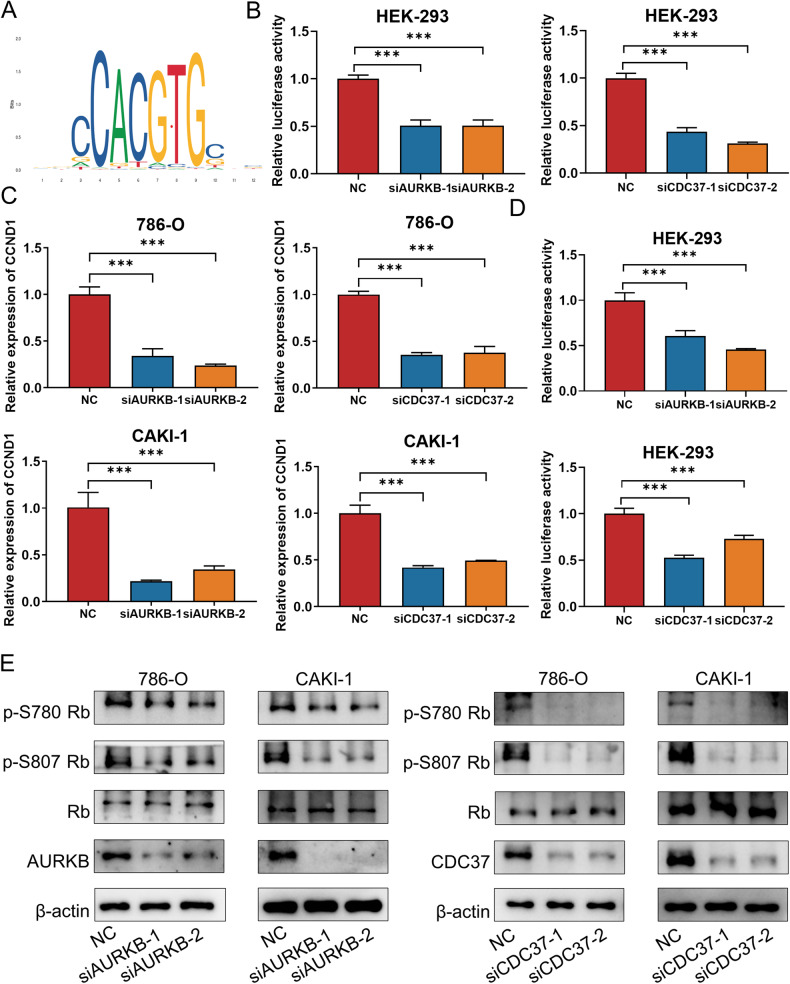
AURKB/CDC37 regulates MYC transcriptional program and promotes the phosphorylation of Rb1. **A** DNA-binding motif of MYC (JASPAR). **B** Luciferase assays were performed in HEK-293 cells co-transfected with siNC/siAURKB/siCDC37 and MYC motif. **C** CCND1 mRNA levels in 786-O and CAKI-1 cells with AURKB or CDC37 knockdown were detected by qRT-PCR. **D** Luciferase assays were performed in HEK-293 cells co-transfected with siNC/siAURKB/siCDC37 and CCND1 promoter. **E** Western blotting was used to detect the effect of AURKB or CDC37 knockdown on the expression of phosphorylated Rb in 786-O and CAKI-1 cells. *p < 0.05, **p < 0.01, ***p < 0.001.

We predicted that knockdown of AURKB/CDC37 would affect MYC transcriptional program. To test this notion, The sequence of the MYC transcriptional activity motif predicted by the JASPAR website (https://jaspar.elixir.no/) were subcloned into the pGL3 promoter luciferase vector (Fig. 7A). Luciferase reporter analysis showed that compared with siNC, knockdown of AURKB or CDC37 significantly decreased the luciferase activity, indicating that AURKB and CDC37 positively regulate the transcriptional activity of MYC on their downstream genes (Fig. 7B). We further authenticate our point of view with published, validated MYC-regulated gene CCDN1 [26]. Decreased CCND1 mRNA was observed in 786-O and CAKI-1 cells with AURKB or CDC37 knockdown (Fig. 7C). Subsequently, luciferase assays were performed to determine whether AURKB or CDC37 affected the transcription of MYC target gene CCND1. The sequence of the CCND1 promoter region were subcloned into the pGL3 promoter luciferase vector. As shown in Fig. 7D, The luciferase activity in siAURKB/siCDC37 group was significantly lower than that of siNC. Since AURKB, MYC, and CCND1 expression are highly correlated with the cell cycle, we subsequently investigated the regulatory relationship of their expression in synchronous cells. As shown in Fig. S8A, knockdown of AURKB in synchronized ccRCC cells resulted in down-regulation of MYC protein levels. In synchronous ccRCC cells with CDC37 knockdown, significant protein down-regulation of both AURKB and MYC were observed. (Fig. S8B). Meanwhile, knockdown of AURKB or CDC37 significantly downregulated CCND1 mRNA in synchronized ccRCC cells (Fig. S78C, D). Taken together, these data suggest that AURKB and CDC37 regulate MYC transcriptional program and in turn promote CCND1 transcription.

Previous studies have shown that CyclinD1 plays a very important role in regulating Rb phosphorylation, and the phosphorylation and non-phosphorylation of Rb determine the activity of E2F1 [27]. We have shown that depletion of AURKB or CDC37 in ccRCC cells decreases CCND1 transcription, so does it further affect Rb phosphorylation as well as E2F1 release? A reduction of p-S780 Rb and p-S807 Rb were observed with AURKB or CDC37 knockdown in 786-O and CAKI-1 cells (Fig. 7E). These results suggest that AURKB increases Rb phosphorylation and promotes E2F1 release.

### E2F1 promotes ccRCC progression by regulating AURKB transcription

**Fig. 8:**
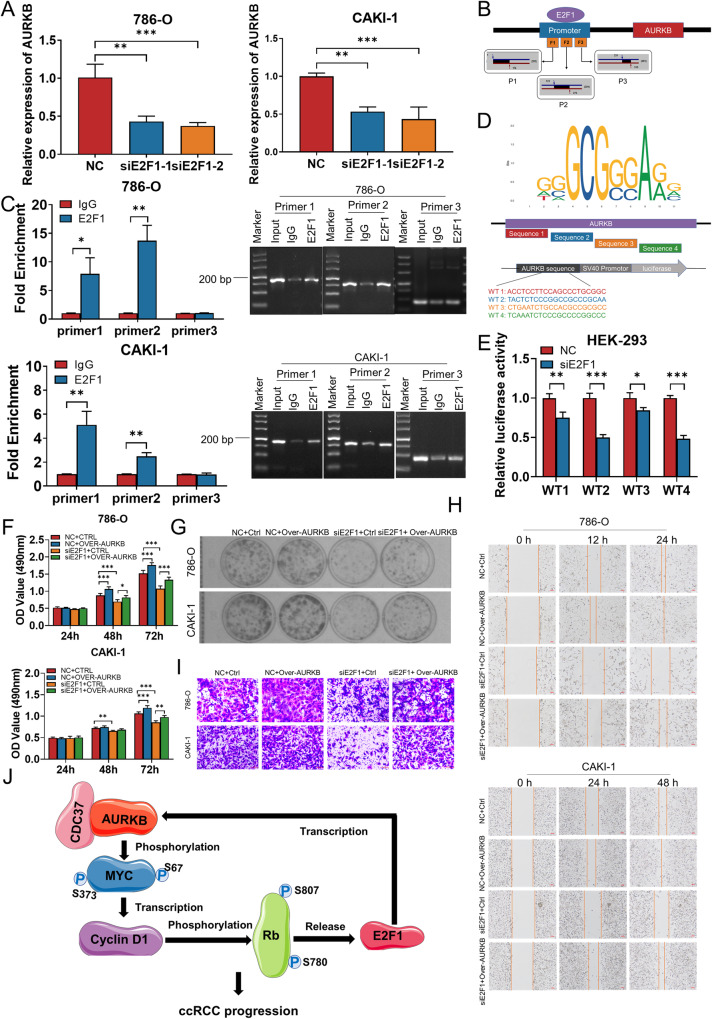
E2F1 activates AURKB expression directly. **A** AURKB mRNA levels in 786-O and CAKI-1 cells with E2F1 knockdown were detected by qRT-PCR. **B** Schematic diagram of primer design for ChIP-qRT-PCR in the binding site between E2F1 and the promoter region of AURKB. **C** The binding relationship between E2F1 and the promoter region of AURKB was verified by ChIP-qRT-PCR and agarose gel electrophoresis. **D** The binding sequence of E2F1 on AURKB predicted by JASPAR was subcloned into the pGL3 promoter luciferase vector. **E** Luciferase assays were performed in HEK-293 cells co-transfected with siNC/siE2F1-2 and AURKB promoter. **F**, **G** MTT and colony formation assays were performed to determine the impact of cell viability treated with NC+ Ctrl, NC + Over-AURKB, siE2F1-2+Ctrl, siE2F1-2+Over-AURKB in ccRCC cells. **H**, **I** Transwell and wound‐healing analysis represented the migration and metastasis capacity of ccRCC cells co‐transfected with NC + Ctrl, NC + Over-AURKB, siE2F1-2 + Ctrl, siE2F1-2 + Over-AURKB. **J** The schematic illustrates that CDC37 and AURKB complexes directly phosphorylate MYC, enhance MYC stability and transcriptional activity, promote Rb phosphorylation and E2F1 release, and in turn activate the transcription of AURKB, forming an AURKB/E2F1 positive feedforward loop that promotes ccRCC progression. *p < 0.05, **p < 0.01, ***p < 0.001.

Since E2F1 is a transcription factor, we considered whether it acts as a transcription factor that in turn regulates AURKB transcription, and interestingly, according to predictions on the UCSC website (https://genome.ucsc.edu/), E2F1 may bind in the promoter region of AURKB. Meanwhile, E2F1 and AURKB expression showed a significant positive correlation in ccRCC (*r* = 0.72, Fig. S9A). To assess the potential role of E2F1 in regulating AURKB, we knocked down E2F1 in 786-O and CAKI-1 cells by siRNAs (Fig. S9B). Depletion of E2F1 reduced AURKB at the mRNA level, suggesting E2F1 could promote the expression of AURKB (Fig. 8A). The binding sequences of E2F1to AURKB promoter regions were searched based on the UCSC database (https://genome.ucsc.edu/), and primers were designed segmentally according to the sequences to verify whether E2F1 binds to AURKB promoter regions (Fig. 8B). Subsequently, ChIP was performed with E2F1 antibody, and the extracted DNA was detected by qRT-PCR using the above primers and then subjected to agarose gel electrophoresis. The results suggested that E2F1 significantly bound to site 1 and 2 of the AURKB promoter region, which was further confirmed by agarose gel electrophoresis (Fig. 8C). The site 1 and 2 sequence were further used to predict the binding site of E2F1 on AURKB using the JASPAR website (https://jaspar.elixir.no/), and the predicted binding sequence was subcloned into the pGL3 promoter luciferase vector (Fig. 8D). Luciferase reporter analysis showed that knockdown of E2F1 could decrease luciferase activity of vector in HEK-293 cells (Fig. 8E). The above results consistently indicated that E2F1 bound to the AURKB promoter region and activate AURKB transcription.

In addition, E2F1 siRNA and AURKB overexpression plasmids were co-transfected into 786-0 and CAKI-1 cells to assess whether enhanced AURKB expression could rescue the inhibitory effects of E2F1 depletion on the proliferation and migration of ccRCC cells. MTT assay showed that E2F1 knockdown inhibited ccRCC cell proliferation, while AURKB overexpression partially reversed the inhibitory effect of E2F1 knockdown on ccRCC cell proliferation (Fig. 8F). Consistent with the MTT results, the decrease in cell clone formation ability of ccRCC cells caused by knockdown of E2F1 was also restored by co-transfection of AURKB overexpression (Fig. 8G and Fig. S9C). Additionally, increased expression of AURKB induced migration of 786-O and CAKI-1 cells, while knockdown of E2F1 reduced these effects of AURKB on ccRCC cells after co‐transfection of siE2F1 and AURKB overexpression plasmids (Fig. 8H-I and Fig. S9D-E).

Based on these findings, CDC37 and AURKB complexes directly phosphorylate MYC, enhance MYC stability and transcriptional activity, promote Rb phosphorylation and E2F1 release, and in turn activate the transcription of AURKB, forming an AURKB/E2F1 positive feedforward loop that promotes ccRCC progression (Fig. 8J).

## Discussion

Tumor development is a complex pathological process involving multiple genetic alterations, including overexpression of oncogenes and/or inactivation of tumor suppressor genes [28]. Screening novel tumor markers and exploring their molecular mechanisms in the occurrence and development of tumors have become the hotspots of current scientific research. RCC as the second largest malignant tumors in the urinary system, to explore the early diagnostic markers and therapeutic targets is imperative [1].

In recent years, bioinformatics, as a science combining molecular biology and information technology, has studied the molecular mechanism of diseases through data mining at the molecular level, and found a large number of tumor markers that can be applied in clinical practice [29]. These findings are of great significance for revealing the molecular mechanism of tumor pathogenesis and improving the early diagnosis and prognosis of tumors. In the current study, we identified AURKB as a novel key gene in ccRCC progression by screening of differentially expressed genes in ccRCC, construction of WGCNA, and machine learning. AURKB has been found to be overexpressed in a variety of cancers and act as an oncogene, which is a potential target for cancer therapy [10]. Consistent with these findings, we found that AURKB is abnormally highly expressed in ccRCC tissues and cell lines and promotes ccRCC cell proliferation and migration in vitro and in vivo, indicating that AURKB acts as an oncogene in ccRCC and can be used as a novel marker for ccRCC.

Numerous studies have shown that AURKB promotes tumor development by phosphorylating downstream substrates to increase their activity or stability. AURKB phosphorylates MCAK at S192, thereby regulating migration and invasion of gastric cancer [30]. In addition, AURKB phosphorylates survivin, a member of the apoptosis suppressor protein (IAP) family, thereby regulating the proliferation of triple-negative breast cancer cells [31]. Another study reported that Aurora B phosphorylates p53 at S183, T211 and S215 to accelerate its degradation, thereby inhibiting the expression of p53 target genes involved in cell cycle arrest and apoptosis to promote tumor development [32]. Therefore, it is imperative to explore potential novel phosphorylation substrates and sites of AURKB to elucidate its molecular mechanism in tumor progression.

MYC are commonly found on the path to cancer [33]. The significance of MYC deregulation has been recognized in ccRCC [34, 35]. We found that silencing AURKB inhibited MYC expression in ccRCC cells, implying that AURKB and MYC are co-expressed in ccRCC cells. In parallel, we identified MYC as a AURKB binding partner by simultaneous immunofluorescence and Co-IP analysis. Although previous studies have proposed that AURKB can directly phosphorylate MYC at S67, importantly we also report another novel site, S373 [36]. A well-defined event in MYC degradation is that after phosphorylation of T58 by GSK3β, MYC is recognized by the E3 ubiquitin ligase FBXW7 and degraded by the 26S proteasome [25, 37]. Our data indicate that knockdown of AURKB significantly reduced the half-life of endogenous MYC in 786-O and CAKI-1 cells. At the same time, MG132 effectively restored the reduced effect of AURKB knockdown on MYC protein abundance, indicating that AURKB knockdown stimulates proteasomal degradation of MYC. In addition, AZD1152 treatment resulted in a significant increase in MYC p-T58 in 786-O and CAKI-1 cells, suggesting inactivation of AURKB enhances GSK3β-mediated T58 phosphorylation. AURKB expression significantly prolonged the half-life of exogenous MYC, from 30 to >60 min, while AURKB deactivation mutant kinase (K106R) failed to prolong its half-life, suggesting that the stability of MYC need AURKB kinase activity. Furthermore, in comparison with the WT counterpart, the mutation of S67D, S373D and S67D S373D increased the half-life of MYC, whereas S67A, S373A and S67A S373A further decreased the MYC half-life. Knockdown of AURKB resulted in a significant increase in the ubiquitination level of MYC WT, but not S67A, S373A and S67A S373A mutants. These data indicated that AURKB-mediated phosphorylation of MYC at S67 and S373 contributes to MYC stability.

CDC37 is an important molecular chaperone of HSP90 and plays a key role in regulating substrate protein kinase stability and activity [38]. Protein kinases catalyzed by CDC37 have been identified including Raf-1, Akt, EGFR, and platelet-derived growth factor receptor kinases [39–42]. Previous studies have tentatively suggested that CDC37 regulates the cell cycle by regulating the stability of Aurora B kinase [43]. Consistent with this study, we observed the interaction between AURKB and CDC37, and CDC37 phenocopy AURKB in ccRCC cells. In addition, CDC37 promotes Aurora B kinase stability which in turn promotes MYC stability in ccRCC. Moreover, we also indicate AURKB/CDC37 complex regulate MYC transcriptional program and in turn promote CCND1 transcription. Previous studies have shown that CyclinD1 plays a very important role in regulating Rb phosphorylation, and the phosphorylation and non-phosphorylation of Rb determine the activity of E2F1 [20, 27, 44, 45]. In our study, a reduction of p-S780 Rb and p-S807 Rb were observed with AURKB knockdown in 786-O and CAKI-1 cells, suggesting that AURKB promotes phosphorylation of Rb and thus facilitates E2F1 release from Rb.

The E2F transcription factor family plays a key role in cell cycle regulation and apoptosis, among which E2F1 is a transcription activator [46]. The transcriptional targets of E2F1 include many cell cycle related proteins such as CDC2, CDC25A, Cyclin D1, and Cyclin E [47]. Our findings suggest that E2F1 bound to the AURKB promoter region and activate AURKB transcription.

In conclusion, our findings indicated that AURKB promotes ccRCC growth and metastasis in vitro and in vivo, and was identified as a novel ccRCC marker. Notably, CDC37 was found to be a kinase molecular chaperone of AURKB and phenocopy AURKB in ccRCC. Mechanistically, AURKB/CDC37 complex mediate the stabilization of MYC protein by directly phosphorylating MYC at S67 and S373 to promote ccRCC development. At the same time, we demonstrated that the AURKB/CDC37 complex activates MYC to transcribe CCND1, enhances Rb phosphorylation, and promotes E2F1 release, which in turn activates AURKB transcription and forms a positive feedforward loop in ccRCC. Overall, our study highlights AURKB plays an important role in ccRCC progression and may represent a promising therapeutic target for ccRCC.

### Supplementary information

Supplementary information

## Data Availability

The data and supporting materials regarding the findings of this study are accessible in the main document as well as in supplemental tables, figures, and additional information. Please contact the corresponding author for data requests.

## References

[1] Sung H, Ferlay J, Siegel RL, Laversanne M, Soerjomataram I, Jemal A (2021). GlobaL Cancer Statistics 2020: GLOBOCAN estimates of incidence and mortality worldwide for 36 cancers in 185 countries. CA Cancer J Clin.

[2] Shuch B, Amin A, Armstrong AJ, Eble JN, Ficarra V, Lopez-Beltran A (2015). Understanding pathologic variants of renal cell carcinoma: distilling therapeutic opportunities from biologic complexity. Eur Urol.

[3] Rini BI, Campbell SC, Escudier B (2009). Renal cell carcinoma. Lancet.

[4] Nerich V, Hugues M, Paillard MJ, Borowski L, Nai T, Stein U (2014). Clinical impact of targeted therapies in patients with metastatic clear-cell renal cell carcinoma. Onco Targets Ther.

[5] Motzer RJ, Bukowski RM, Figlin RA, Hutson TE, Michaelson MD, Kim ST (2008). Prognostic nomogram for sunitinib in patients with metastatic renal cell carcinoma. Cancer.

[6] Siegel RL, Miller KD, Jemal A (2017). Cancer statistics, 2017. CA Cancer J Clin.

[7] Glover DM, Leibowitz MH, McLean DA, Parry H (1995). Mutations in aurora prevent centrosome separation leading to the formation of monopolar spindles. Cell.

[8] Katayama H, Brinkley WR, Sen S (2003). The Aurora kinases: role in cell transformation and tumorigenesis. Cancer Metastasis Rev.

[9] Yan M, Wang C, He B, Yang M, Tong M, Long Z (2016). Aurora-A kinase: a potent oncogene and target for cancer therapy. Med Res Rev.

[10] Portella G, Passaro C, Chieffi P (2011). Aurora B: a new prognostic marker and therapeutic target in cancer. Curr Med Chem.

[11] Stewart S, Fang G (2005). Destruction box-dependent degradation of aurora B is mediated by the anaphase-promoting complex/cyclosome and Cdh1. Cancer Res.

[12] Zeitlin SG, Shelby RD, Sullivan KF (2001). CENP-A is phosphorylated by Aurora B kinase and plays an unexpected role in completion of cytokinesis. J Cell Biol.

[13] Shimada M, Goshima T, Matsuo H, Johmura Y, Haruta M, Murata K (2016). Essential role of autoactivation circuitry on Aurora B-mediated H2AX-pS121 in mitosis. Nat Commun.

[14] Tang A, Gao K, Chu L, Zhang R, Yang J, Zheng J (2017). Aurora kinases: novel therapy targets in cancers. Oncotarget.

[15] Ding L, Yang L, He Y, Zhu B, Ren F, Fan X (2018). CREPT/RPRD1B associates with Aurora B to regulate Cyclin B1 expression for accelerating the G2/M transition in gastric cancer. Cell Death Dis.

[16] Subramaniyan B, Kumar V, Mathan G (2017). Effect of sodium salt of Butrin, a novel compound isolated from Butea monosperma flowers on suppressing the expression of SIRT1 and Aurora B kinase-mediated apoptosis in colorectal cancer cells. Biomed Pharmacother.

[17] Belluti S, Rigillo G, Imbriano C (2020). Transcription factors in cancer: when alternative splicing determines opposite cell fates. Cells.

[18] Ren B, Cam H, Takahashi Y, Volkert T, Terragni J, Young RA (2002). E2F integrates cell cycle progression with DNA repair, replication, and G(2)/M checkpoints. Genes Dev.

[19] Helin K, Harlow E, Fattaey A (1993). Inhibition of E2F-1 transactivation by direct binding of the retinoblastoma protein. Mol Cell Biol.

[20] Engeland K (2022). Cell cycle regulation: p53-p21-RB signaling. Cell Death Differ.

[21] Yu H, Li Z, Wang M (2020). Expression and prognostic role of E2F transcription factors in high-grade glioma. CNS Neurosci Ther.

[22] Chen S, He Z, Peng T, Zhou F, Wang G, Qian K, (2021). PRR11 promotes ccRCC tumorigenesis by regulating E2F1 stability. JCI Insight.

[23] Xu TP, Wang YF, Xiong WL, Ma P, Wang WY, Chen WM (2017). E2F1 induces TINCR transcriptional activity and accelerates gastric cancer progression via activation of TINCR/STAU1/CDKN2B signaling axis. Cell Death Dis.

[24] Li F, Feng Y, Jiang Q, Zhang J, Wu F, Li Q (2022). Pan-cancer analysis, cell and animal experiments revealing TEAD4 as a tumor promoter in ccRCC. Life Sci.

[25] Gregory MA, Qi Y, Hann SR (2003). Phosphorylation by glycogen synthase kinase-3 controls c-myc proteolysis and subnuclear localization. J Biol Chem.

[26] Yang Z, Xu T, Xie T, Yang L, Wang G, Gao Y (2022). CDC42EP3 promotes glioma progression via regulation of CCND1. Cell Death Dis.

[27] Mandigo AC, Yuan W, Xu K, Gallagher P, Pang A, Guan YF (2021). RB/E2F1 as a master regulator of cancer cell metabolism in advanced disease. Cancer Discov.

[28] Hanahan D, Weinberg RA (2011). Hallmarks of cancer: the next generation. Cell.

[29] Jiang P, Sinha S, Aldape K, Hannenhalli S, Sahinalp C, Ruppin E (2022). Big data in basic and translational cancer research. Nat Rev Cancer.

[30] Ritter A, Sanhaji M, Friemel A, Roth S, Rolle U, Louwen F (2015). Functional analysis of phosphorylation of the mitotic centromere-associated kinesin by Aurora B kinase in human tumor cells. Cell Cycle.

[31] Garlapati C, Joshi S, Bhattarai S, Krishnamurthy J, Turaga RC, Nguyen T (2023). PLK1 and AURKB phosphorylate survivin differentially to affect proliferation in racially distinct triple-negative breast cancer. Cell Death Dis.

[32] Gully CP, Velazquez-Torres G, Shin JH, Fuentes-Mattei E, Wang E, Carlock C (2012). Aurora B kinase phosphorylates and instigates degradation of p53. Proc Natl Acad Sci USA.

[33] Dang CV (2012). MYC on the path to cancer. Cell.

[34] Patel SA, Hirosue S, Rodrigues P, Vojtasova E, Richardson EK, Ge J (2022). The renal lineage factor PAX8 controls oncogenic signalling in kidney cancer. Nature.

[35] Bailey ST, Smith AM, Kardos J, Wobker SE, Wilson HL, Krishnan B (2017). MYC activation cooperates with Vhl and Ink4a/Arf loss to induce clear cell renal cell carcinoma. Nat Commun.

[36] Jiang J, Wang J, Yue M, Cai X, Wang T, Wu C (2020). Direct phosphorylation and stabilization of MYC by Aurora B kinase promote T-cell leukemogenesis. Cancer Cell.

[37] Welcker M, Orian A, Jin J, Grim JE, Harper JW, Eisenman RN (2004). The Fbw7 tumor suppressor regulates glycogen synthase kinase 3 phosphorylation-dependent c-Myc protein degradation. Proc Natl Acad Sci USA.

[38] Gray PJ, Stevenson MA, Calderwood SK (2007). Targeting Cdc37 inhibits multiple signaling pathways and induces growth arrest in prostate cancer cells. Cancer Res.

[39] Silverstein AM, Grammatikakis N, Cochran BH, Chinkers M, Pratt WB (1998). p50(cdc37) binds directly to the catalytic domain of Raf as well as to a site on hsp90 that is topologically adjacent to the tetratricopeptide repeat binding site. J Biol Chem.

[40] Basso AD, Solit DB, Chiosis G, Giri B, Tsichlis P, Rosen N (2002). Akt forms an intracellular complex with heat shock protein 90 (Hsp90) and Cdc37 and is destabilized by inhibitors of Hsp90 function. J Biol Chem.

[41] Lavictoire SJ, Parolin DA, Klimowicz AC, Kelly JF, Lorimer IA (2003). Interaction of Hsp90 with the nascent form of the mutant epidermal growth factor receptor EGFRvIII. J Biol Chem.

[42] Matei D, Satpathy M, Cao L, Lai YC, Nakshatri H, Donner DB (2007). The platelet-derived growth factor receptor alpha is destabilized by geldanamycins in cancer cells. J Biol Chem.

[43] Lange BM, Rebollo E, Herold A, Gonzalez C (2002). Cdc37 is essential for chromosome segregation and cytokinesis in higher eukaryotes. EMBO J.

[44] Komori T (2013). Regulation of Rb family proteins by Cdk6/Ccnd1 in growth plates. Cell Cycle.

[45] Solomon DA, Wang Y, Fox SR, Lambeck TC, Giesting S, Lan Z (2003). Cyclin D1 splice variants. Differential effects on localization, RB phosphorylation, and cellular transformation. J Biol Chem.

[46] Cam H, Dynlacht BD (2003). Emerging roles for E2F: beyond the G1/S transition and DNA replication. Cancer Cell.

[47] Bracken AP, Ciro M, Cocito A, Helin K (2004). E2F target genes: unraveling the biology. Trends Biochem Sci.

