# Supplementary material for: AURKB/CDC37 complex promotes clear cell renal cell carcinoma progression via phosphorylating MYC and constituting an AURKB/E2F1-positive feedforward loop
Source: Cell Death Dis. 2024 Jun 18;15(6):427. doi: 10.1038/s41419-024-06827-y (PMC11189524; doi:10.1038/s41419-024-06827-y)

Supplementary Figures

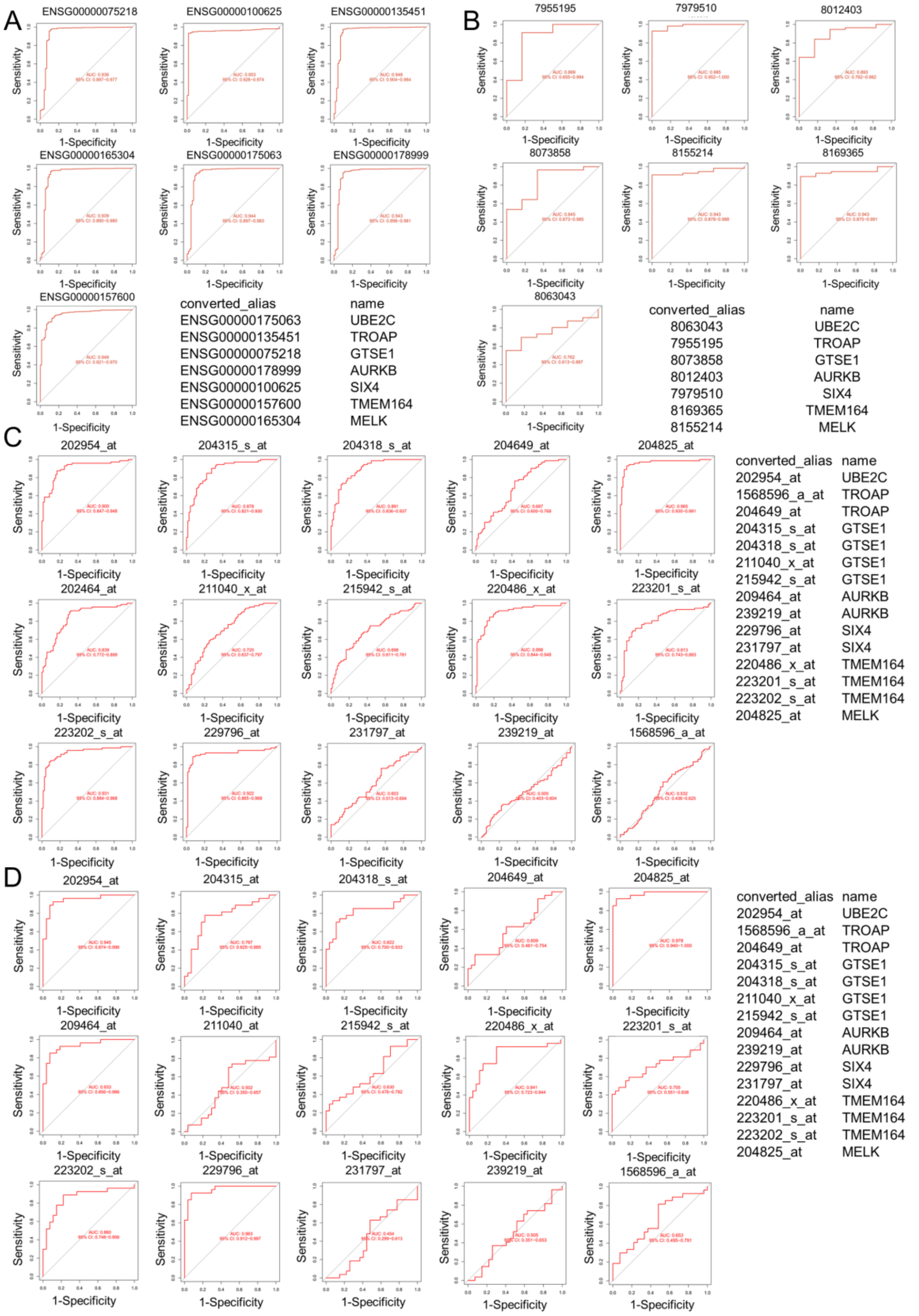

Figure S1. Machine learning to analyze the sensitivity of 7 key genes to ccRCC. The data in TCGA database (A) and three GEO databases, gse53000 (B), gse53757 (C) and gse66272 (D) were used for machine learning to analyze the sensitivity of 7 key genes to ccRCC.

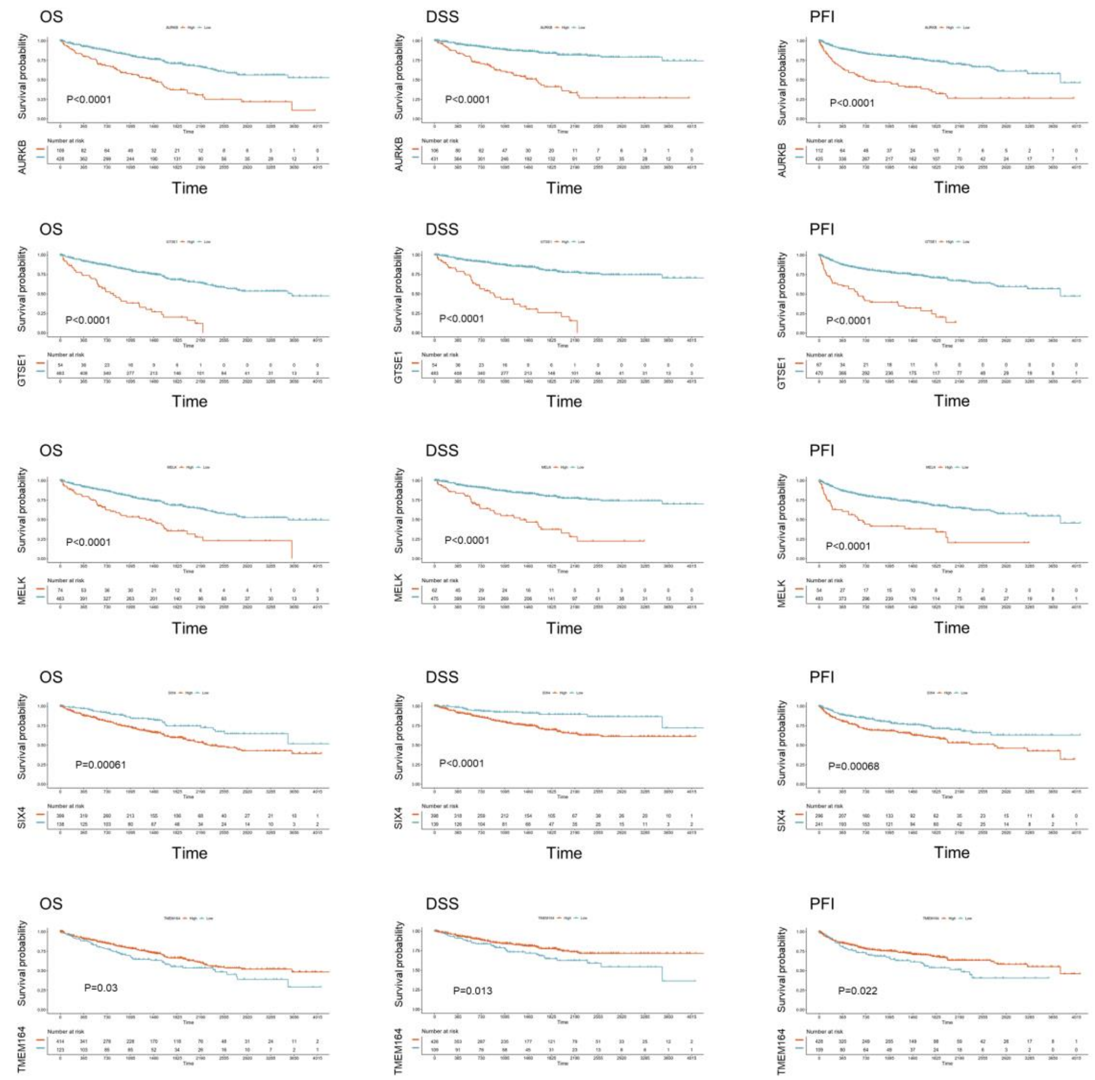

B

OS

	pvalue	Hazard ratio
GTSE1	<0.001	1.077(1.057-1.096)
AURKB	<0.001	1.038(1.028-1.048)
SIX4	0.003	1.075(1.025-1.127)
MELK	<0.001	1.048(1.035-1.060)

DSS

	pvalue	Hazard ratio
GTSE1	<0.001	1.090(1.070-1.111)
AURKB	<0.001	1.043(1.033-1.054)
SIX4	0.002	1.086(1.029-1.145)
MELK	<0.001	1.063(1.047-1.078)

PFI

	pvalue	Hazard ratio
GTSE1	<0.001	1.068(1.049-1.087)
AURKB	<0.001	1.035(1.025-1.044)
MELK	<0.001	1.037(1.026-1.049)

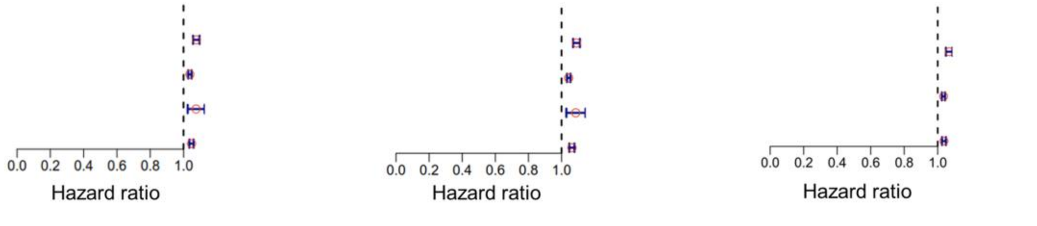

Figure S2. Correlation analysis of key genes with ccRCC survival. (A) TCGA data were used to calculate the influence of key gene expression on prognostic data of ccRCC, including OS, DSS and PFI. (B) Cox regression map was used to investigate the influence of key genes on prognostic types of ccRCC.

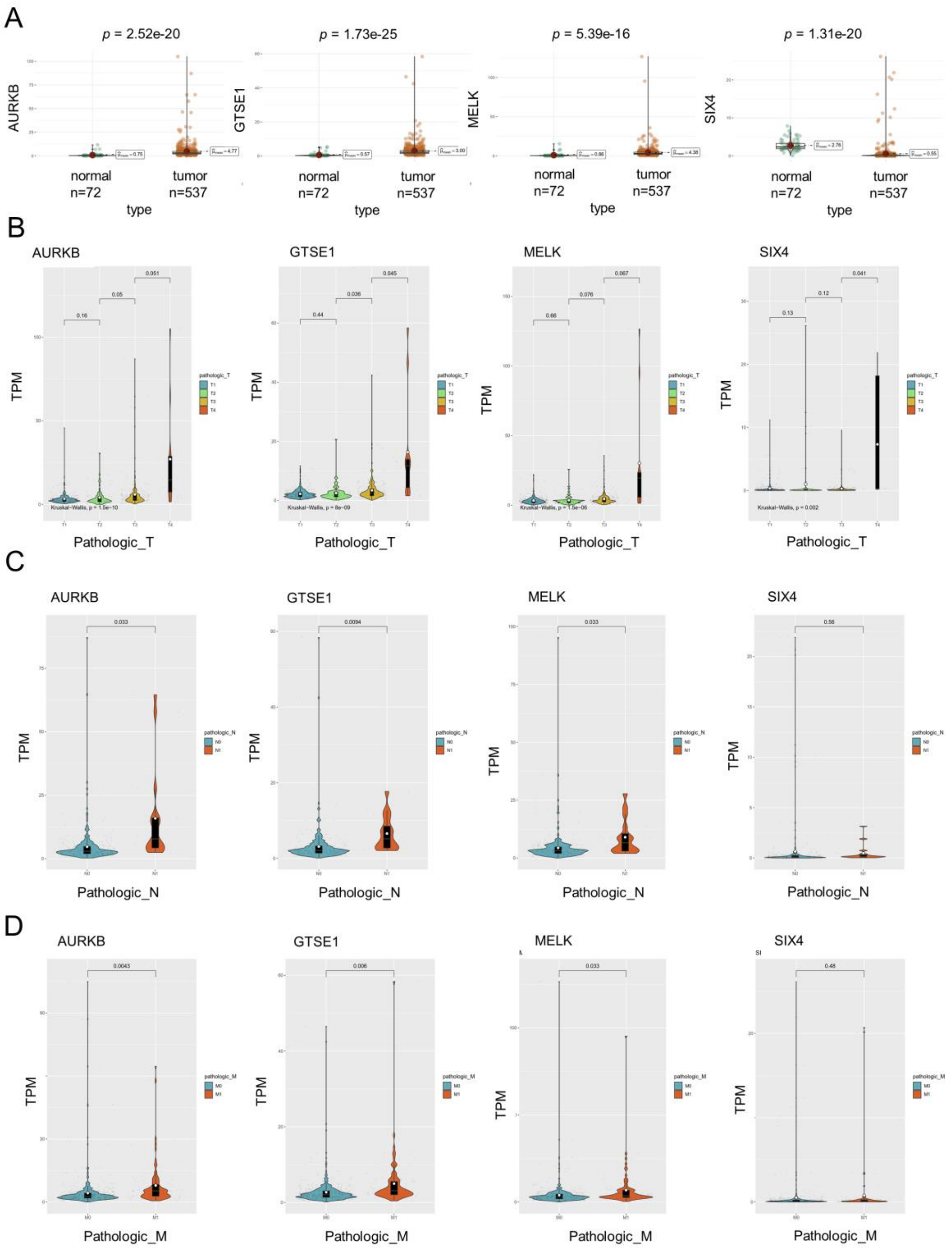

Figure S3. Expression and clinical correlation analysis of key genes. (A) Expression of key genes in ccRCC. The correlation between key genes expression and clinical stage in the TCGA databases. The expression of key genes in different topography (B), lymph node metastasis (C), distant

metastasis (D).

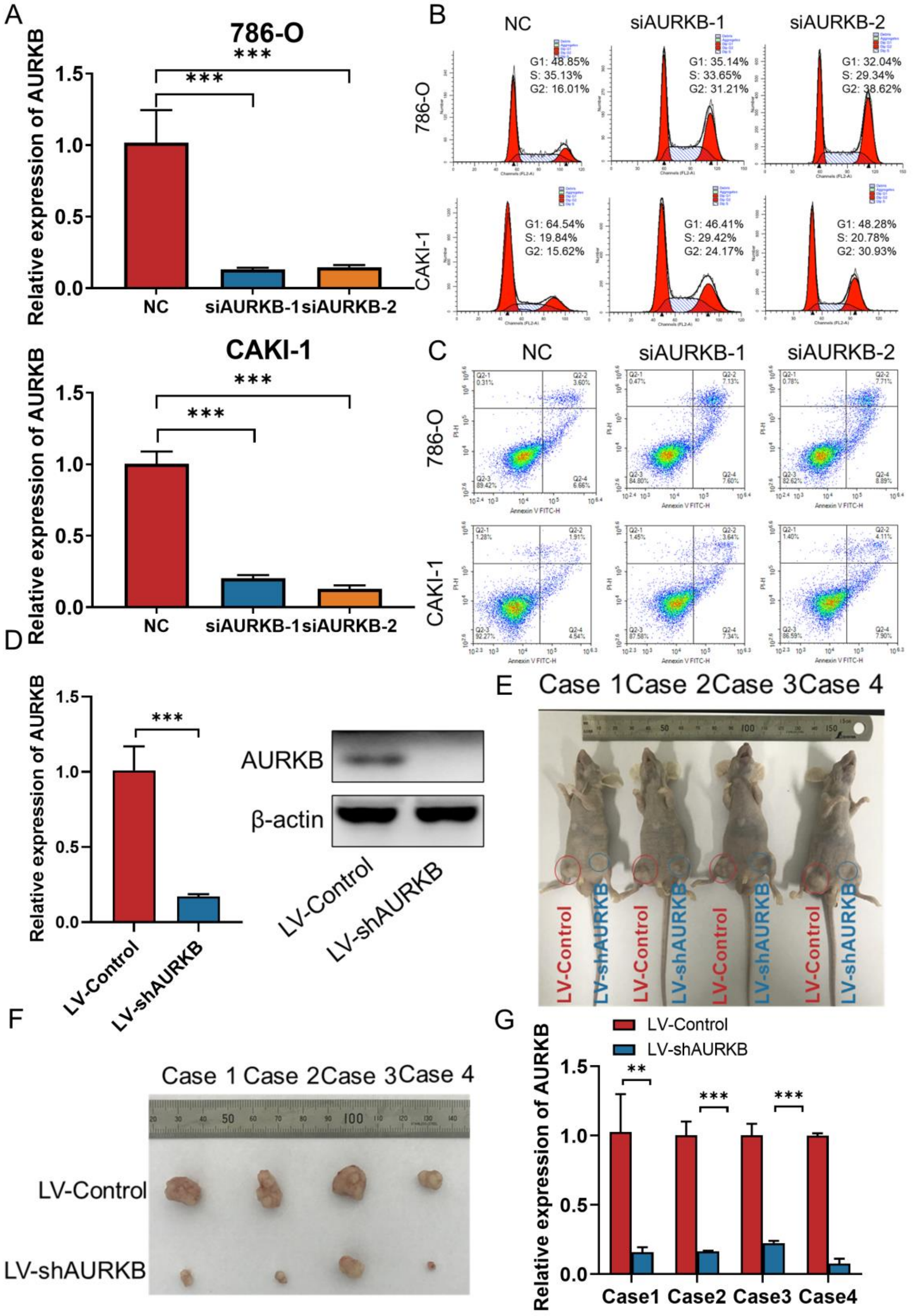

Figure S4. AURKB is up-regulated in ccRCC and promotes the proliferation and migration of ccRCC cells in vitro in vivo. (A) mRNA expression levels of AURKB in 786-O and CAKI-1 cells were detected by qRT-PCR to confirm the knockdown efficiency of both siRNAs. (B) Flow

cytometry was used to detect the effect of AURKB knockdown on cell cycle of 786-O and CAKI-1 cells. (C) Flow cytometry was used to detect the effect of AURKB knockdown on cell apoptosis of 786-O and CAKI-1 cells. (D) qRT-PCR and western blotting were used to detect AURKB mRNA and protein expression in LV-Control and LV-shAURKB cells. (E) Gross morphology of tumors after 28 days of injection of LV-Control and LV-shAURKB cells. (F) Size of xenograft tumors 28 days after injection of LV-Control and LV-shAURKB cells. (G) mRNA levels of AURKB in xenograft tumors were analyzed by qRT-PCR.

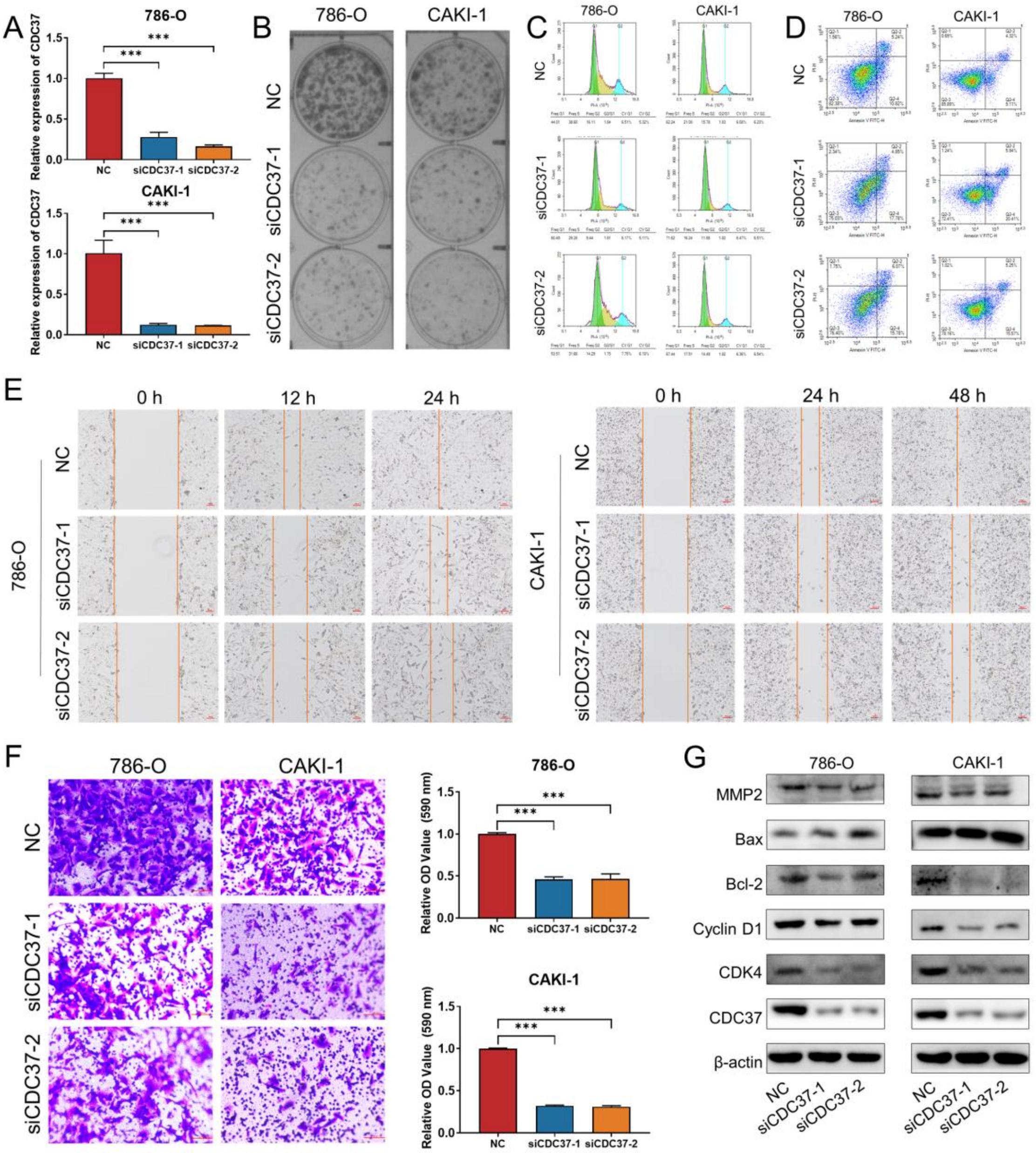

Figure S5. CDC37 phenocopy AURKB in ccRCC. (A) mRNA expression levels of CDC37 in 786-O and CAKI-1 cells were detected by qRT-PCR to confirm the knockdown efficiency of both

siRNAs. (B) Colony formation assay was used to detect the effect of CDC37 knockdown on the colony formation ability of 786-0 and CAKI-1 cells. (C) The effect of CDC37 knockdown on cell cycle of 786-O and CAKI-1 cells was detected by flow cytometry. (D) Flow cytometry was used to detect the effect of CDC37 knockdown on cell apoptosis of 786-O and CAKI-1 cells. (E) Wound healing assay was used to detect the effect of CDC37 knockdown on the migration of 786-O and CAKI-1 cells. (F) Transwell assay was used to detect the effect of CDC37 knockdown on the migration of 786-O and CAKI-1 cells, and the OD value of the traversed cells was used for statistical mapping. (G) Western blotting was used to detect the effect of CDC37 knockdown on the expression of cell cycle, apoptosis and migration related molecules at protein level in 786-O and CAKI-1. *$p < 0.05$, **$p < 0.01$, ***$p < 0.001$.

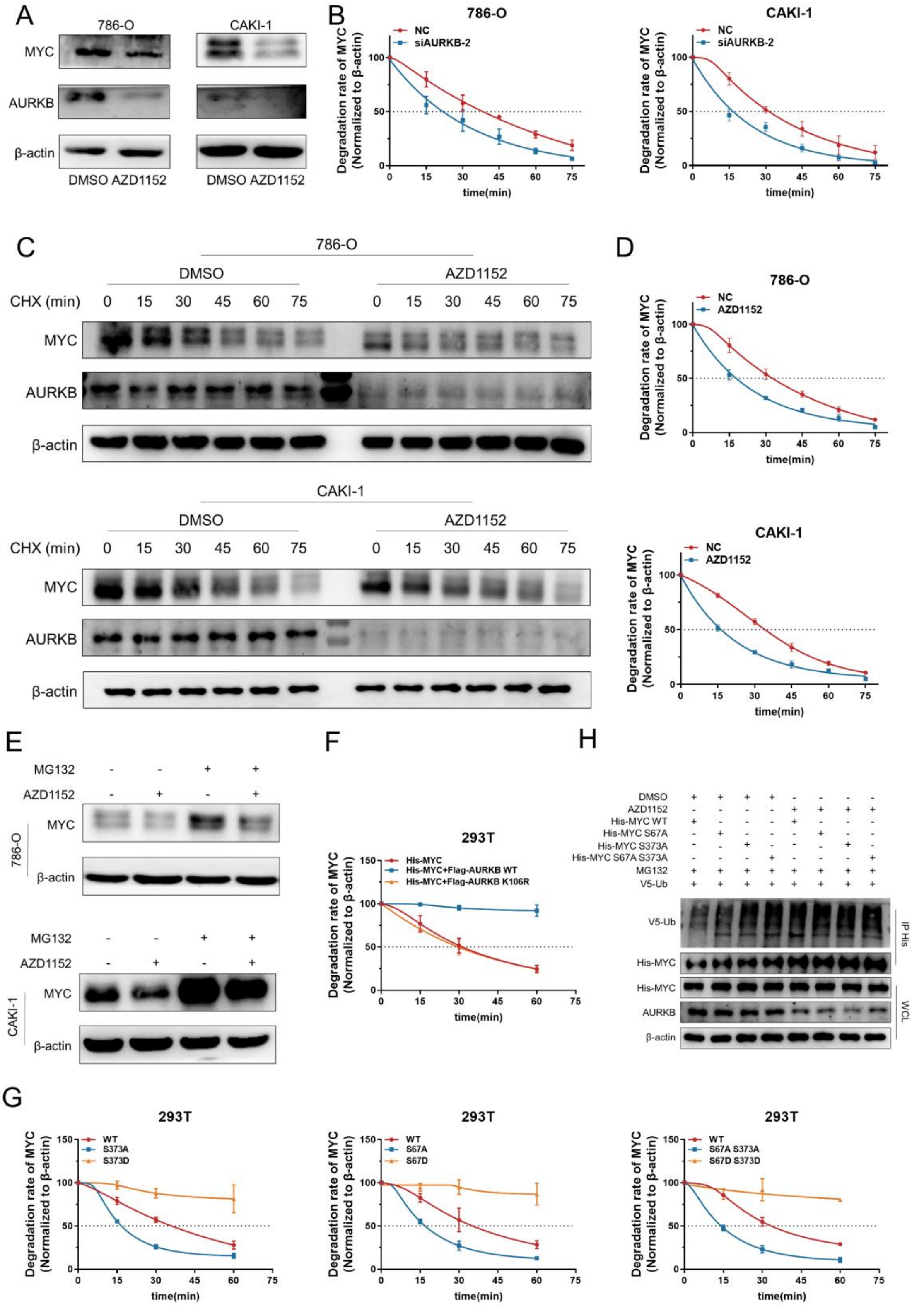

Figure S6. AURKB-mediated MYC phosphorylation contributes to MYC stability. (A) Western blotting was used to detect MYC protein in 786-O and CAKI-1 cells treated with AZD1152 (786-O: 500 nM; CAKI-1: 300 nM). (B) MYC proteins were quantified and plotted. (C) Time course analysis of MYC protein level in 786-O and CAKI-1 cells treated with AZD1152 (786-O: 500 nM;

CAKI-1: 300 nM). (D) MYC proteins were quantified and plotted. (E) 786-O and AKI-1 cells treated with AZD1152 (786-O: 500 nM; CAKI-1: 300 nM) were added to MG132 (10 μM) for 6 h before harvest. AURKB and MYC were analyzed by immunoblot, with β-actin as a control. (F) MYC proteins were quantified and plotted. (G) MYC proteins were quantified and plotted. (H) HEK-293T cells were co-transfected with V5-UB, AZD1152 (0 or 200nM) and His-MYC (WT, S67A, S373A, or S67A S373A), and treated with MG132 (10 μM) for 6 h before harvest. Cell lysates were subjected to Co-IP, ubiquitination, and immunoblot assays.

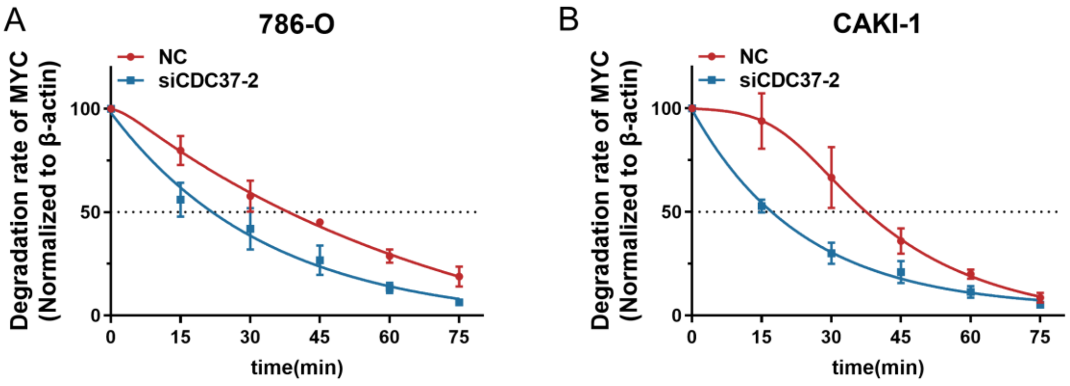

Figure S7. MYC proteins were quantified and plotted. (A) MYC proteins were quantified and plotted in 786-O cells. (B) MYC proteins were quantified and plotted in CAKI-1cells.

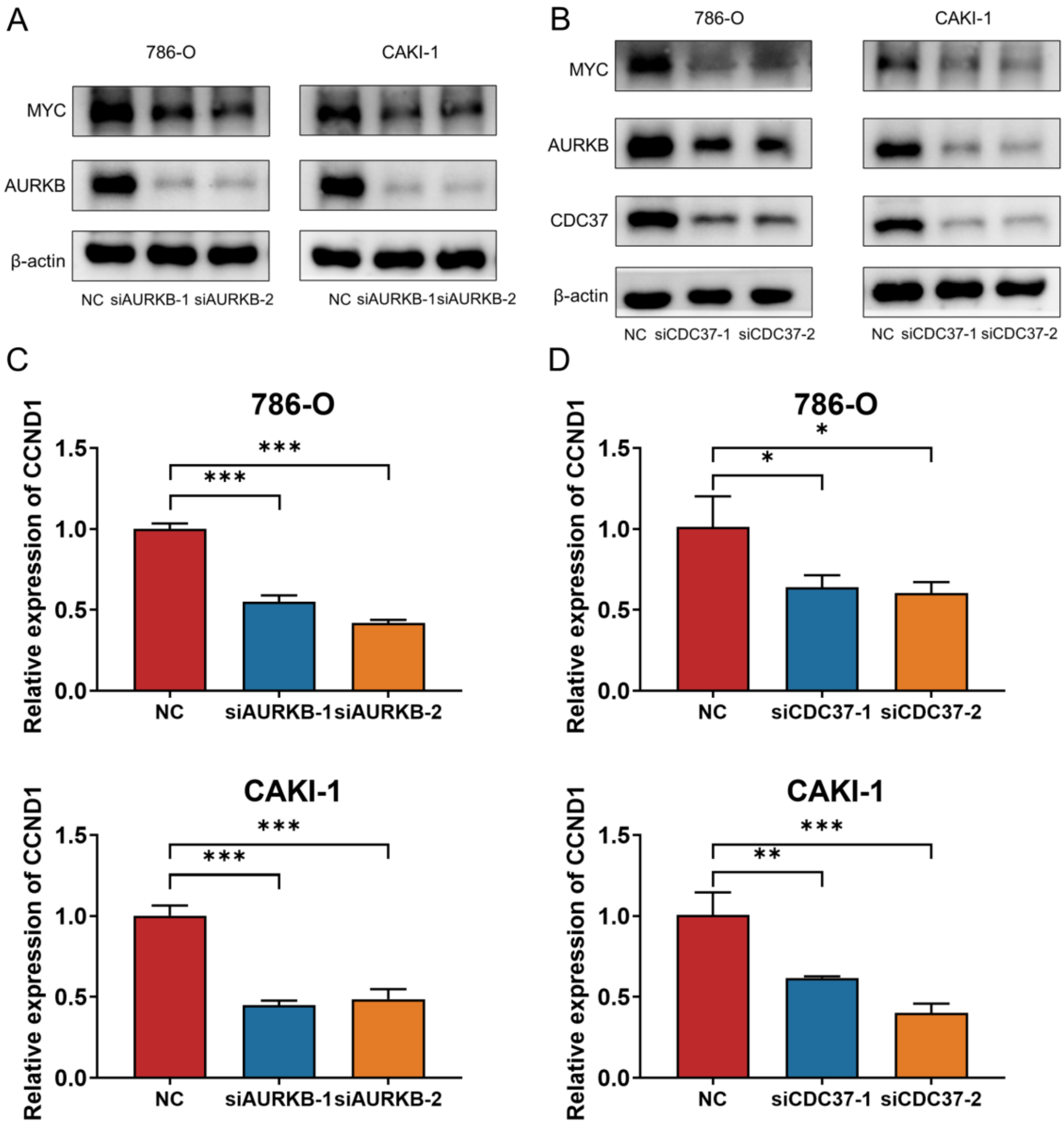

Figure S8. Regulation of CDC37, AURKB, MYC and CCND1 expression in synchronized cells. (A) Western blotting was used to detect the expression changes of MYC after AURKB knockdown in synchronized ccRCC cells. (B) Western blotting was used to detect the expression changes of AURKB and MYC after CDC37 knockdown in synchronized ccRCC cells. (C) CCND1 mRNA levels in synchronized 786-O and CAKI-1 cells with AURKB knockdown were detected by qRT-PCR. (D) CCND1 mRNA levels in synchronized 786-O and CAKI-1 cells with CDC37 knockdown were detected by qRT-PCR. *$p < 0.05$, **$p < 0.01$, ***$p < 0.001$.

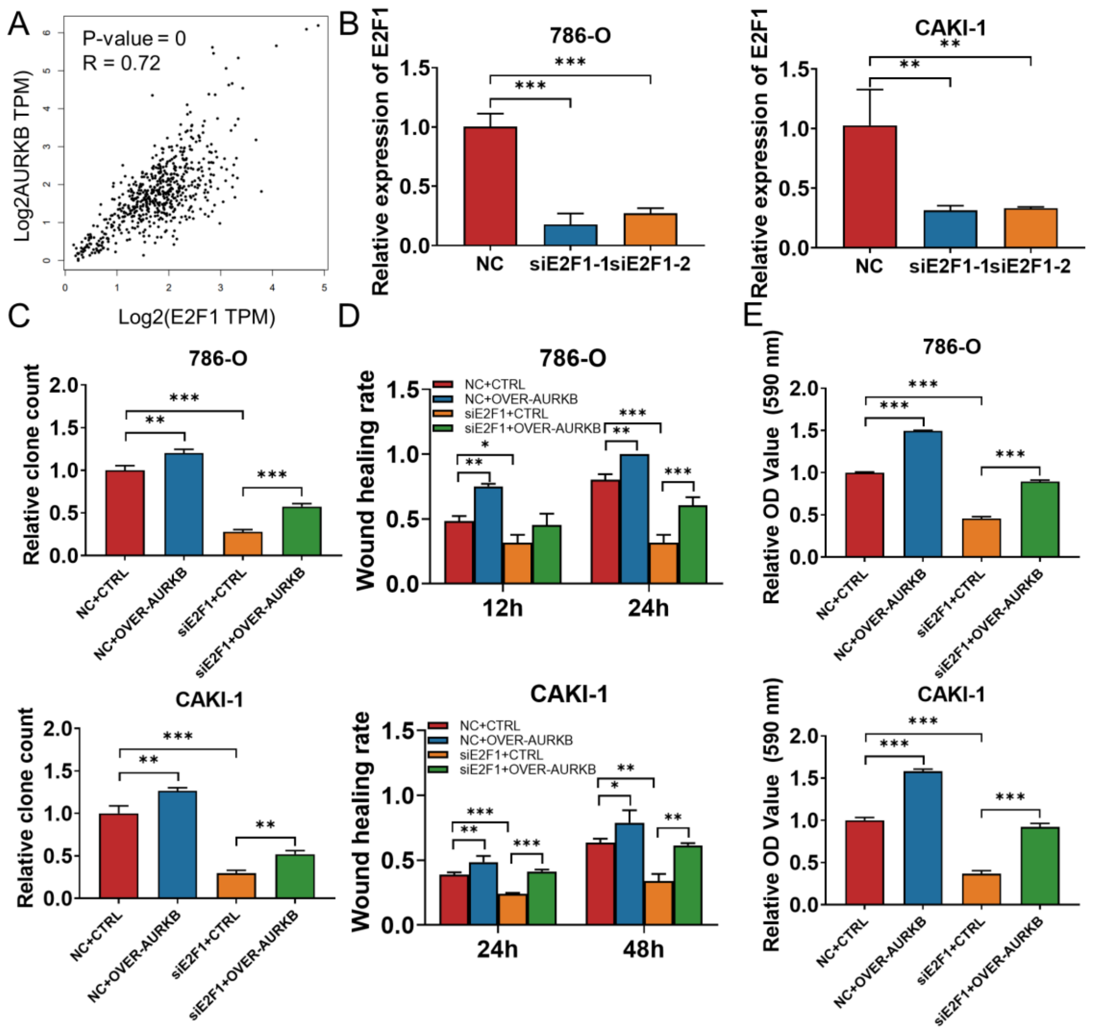

Figure S9. E2F1 activates AURKB expression directly. (A) The relationship between E2F1 and AURKB was assayed by Pearson's r. (B) The mRNA expression levels of E2F1 in 786-O and CAKI-1 cells were detected by qRT-PCR to confirm the knockdown efficiency of both siRNAs. (C) Quantification of colony formation assay in 786-O and CAKI-1 cells after co-transfection with NC+Ctrl, NC+Over-AURKB, siE2F1-2+Ctrl, siE2F1-2+Over-AURKB. (D) Quantification of wound-healing assay in 786-O and CAKI-1 cells after co-transfection with NC+Ctrl, NC+Over-AURKB, siE2F1-2+Ctrl, siE2F1-2+Over-AURKB. (E) Quantification of Transwell assay in 786-O and CAKI-1 cells after co-transfection with NC+Ctrl, NC+Over-AURKB, siE2F1-2+Ctrl, siE2F1-2+Over-AURKB. *$p < 0.05$, **$p < 0.01$, ***$p < 0.001$.

Table S1. DATA and R language description

1. **TCGA data download website and citation**

website: TCGA data download from UCSC XENA (https://xena.ucsc.edu)

Cite:

The UCSC Xena platform for public and private cancer genomics data visualization and interpretation

Mary Goldman, Brian Craft, Mim Hastie, Kristupas Repečka, Fran McDade, Akhil Kamath, Ayan Banerjee, Yunhai Luo, Dave Rogers, View ORCID ProfileAngela N. Brooks, Jingchun Zhu, David Haussler doi: https://doi.org/10.1101/326470

2. **The dataset of clinical stage：**

dataset: phenotype - Phenotypes

hub: https://tcga.xenahubs.net

dataset ID: TCGA.KIRC.sampleMap/KIRC_clinicalMatrix

download: https://tcga-xena-hub.s3.us-east-1.amazonaws.com/download/TCGA.KIRC.sampleMap/KIRC_clinicalMatrix; Full metadata

samples: 945

version: 2019-12-06

type of data: phenotype

raw data: https://tcga data.nci.nih.gov/tcgafiles/ftp_auth/distro_ftpusers/anonymous/tumor/lihc/bcr/

Notes of different clinical stage in manuscript

neoplasm_histologic_grade: G1 G2 G3 G4

pathologic_stage: Stage I Stage II Stage III Stage IV

pathologic_T: T1 T2 T3 T4

pathologic_M: M0 M1

pathologic_N: N0 N1

3. **The survival dataset：**

dataset: phenotype - Curated survival data

hub: https://tcga.xenahubs.net

Curated survival data from the Pan-cancer Atlas paper titled "An Integrated TCGA Pan-Cancer Clinical Data Resource (TCGA-CDR) to drive high quality survival outcome analytics". The paper highlights four types of carefully curated survival endpoints, and recommends the use of the endpoints of OS(overall survial), PFI(progression-free interval), DFI(disease-free interval), and DSS(disease-specific survival) for each TCGA cancer type.

dataset ID: survival/KIRC_survival.txt

download: https://tcga.xenahubs.net/download/survival/KIRC_survival.txt.gz; Full metadata

samples: 944

version: 2018-09-13

type of data: phenotype

raw data: http://www.cell.com/cell/fulltext/S0092-8674(18)30229-0

4. **The version and citation of limma**

Version: 3.54.1

Cite:

Ritchie, M.E., Phipson, B., Wu, D., Hu, Y., Law, C.W., Shi, W., and Smyth, G.K. (2015). limma powers differential expression analyses for RNA-sequencing and microarray studies. Nucleic Acids Research 43(7), e47.

5. **Tthe version and citation of DESeq2**

Version: 1.38.3

Cite:

Love, M.I., Huber, W., Anders, S. Moderated estimation of fold change and dispersion for RNA-seq data with DESeq2 Genome Biology 15(12):550 (2014).

6. **The version and citation of edgeR**

Version: 3.40.2

Cite:

Robinson MD, McCarthy DJ and Smyth GK (2010). edgeR: a Bioconductor package for differential expression analysis of digital gene expression data. Bioinformatics 26,139-140.

McCarthy DJ, Chen Y and Smyth GK (2012). Differential expression analysis of multifactor RNA-Seq experiments with respect to biological variation. Nucleic Acids Research 40,4288-4297.

Chen Y, Lun ATL, Smyth GK (2016). From reads to genes to pathways: differential expression analysis of RNA-Seq experiments using Rsubread and the edgeR quasi-likelihood pipeline. F1000Research 5, 1438.

7. **The version and citation of tidyverse**

Version: 1.3.2

Cite:

Wickham H, Averick M, Bryan J, Chang W, McGowan LD, François R, Grolemund G, Hayes A, Henry L, Hester J, Kuhn M, Pedersen TL, Miller E, Bache SM, Müller K, Ooms J, Robinson D, Seidel DP, Spinu V, Takahashi K, Vaughan D, Wilke C, Woo K, Yutani H (2019). “Welcome to the tidyverse.” _Journal of Open Source Software_, *4*(43), 1686. doi:10.21105/joss.01686 <https://doi.org/10.21105/joss.01686>.

8. **The version and citation of ggplot2**

Version: 3.4.1

Cite:

H. Wickham. ggplot2: Elegant Graphics for Data Analysis. Springer-Verlag New York, 2016.

9. **The version and citation of data.table**

Version: 1.14.8

Cite:

Dowle M, Srinivasan A (2023). _data.table: Extension of `data.frame`_. R package version 1.14.8, <https://CRAN.R-project.org/package=data.table>.

10. **The version and citation of WGCNA**

Version: 1.72.1

Cite:

Langfelder P and Horvath S, WGCNA: an R package for weighted correlation network analysis. BMC Bioinformatics 2008, 9:559 doi:10.1186/1471-2105-9-559

Peter Langfelder, Steve Horvath (2012). Fast R Functions for Robust Correlations and Hierarchical

Clustering. Journal of Statistical Software, 46(11), 1-17. URL http://www.jstatsoft.org/v46/i11/.

11. The version and citation of e1071

Version: 1.7.13

Cite:

Meyer D, Dimitriadou E, Hornik K, Weingessel A, Leisch F (2023). _e1071: Misc Functions of the Department of Statistics, Probability Theory Group (Formerly: E1071), TU Wien_. R package version 1.7-13, <https://CRAN.R-project.org/package=e1071>.

12. The version and citation of kernlab

Version: 0.9.32

Cite:

Karatzoglou A, Smola A, Hornik K (2023). _kernlab: Kernel-Based Machine Learning Lab_. R package version 0.9-32, <https://CRAN.R-project.org/package=kernlab>.

Karatzoglou A, Smola A, Hornik K, Zeileis A (2004). "kernlab - An S4 Package for Kernel Methods in R." _Journal of Statistical Software_, *11*(9), 1-20. doi:10.18637/jss.v011.i09 <https://doi.org/10.18637/jss.v011.i09>.

13. The version and citation of caret

Version: 6.0.93

Cite:

Kuhn M (2022). _caret: Classification and Regression Training_. R package version 6.0-93, <https://CRAN.R-project.org/package=caret>.

14. The version and citation of gbm

Version: 2.1.8.1

Cite:

Greenwell B, Boehmke B, Cunningham J, Developers G (2022). _gbm: Generalized Boosted Regression Models_. R package version 2.1.8.1, <https://CRAN.R-project.org/package=gbm>.

15. The version and citation of randomForest

Version: 4.7.1.1

Cite:

A. Liaw and M. Wiener (2002). Classification and Regression by randomForest. R News 2(3), 18--22.

16. The version and citation of rms

Version: 6.5.0

Cite:

Harrell Jr FE (2023). _rms: Regression Modeling Strategies_. R package version 6.5-0, <https://CRAN.R-project.org/package=rms>.

17. The version and citation of regplot

Version: 1.1

Cite:

Marshall R (2020). _regplot: Enhanced Regression Nomogram Plot_. R package version 1.1, <https://CRAN.R-project.org/package=regplot>.

18. The version and citation of ggstatsplot

Version: 0.11.0

Cite:

Patil, I. (2021). Visualizations with statistical details: The 'ggstatsplot' approach. Journal of Open

Source Software, 6(61), 3167, doi:10.21105/joss.03167.

19. The version and citation of glmnet

Version: 4.1.6

Cite:

Jerome Friedman, Trevor Hastie, Robert Tibshirani (2010). Regularization Paths for Generalized Linear Models via Coordinate Descent. Journal of Statistical Software, 33(1), 1-22. URL https://www.jstatsoft.org/v33/i01/.

Noah Simon, Jerome Friedman, Trevor Hastie, Rob Tibshirani (2011). Regularization Paths for Cox's Proportional Hazards Model via Coordinate Descent. Journal of Statistical Software, 39(5), 1-13. URL https://www.jstatsoft.org/v39/i05/.

Table S2 siRNAs, primers, antibodies and inhibitors used in this study.

Sequences of interference oligonucleotides

Name	Sequence
siNC-sense	UUCUCCGAACGUGUCACGU
siNC-antisense	ACGUGACACGUUCGGAGAA
siAURKB-1 sense	CGCGGCACUUCACAAUUGA
siAURKB-1 antisense	UCAAUUGUGAAGUGCCGCG
siAURKB-2 sense	UUUAGGUCCACCUUGACGAUGCGGC
siAURKB-2 antisense	GCCGCAUCGUCAAGGUGGACCUAAA
siCDC37-1 sense	CGUGGACACGCUCAGCAAA
siCDC37-1 antisense	UUUGCUGAGCGUGUCCACG
siCDC37-2 sense	GUACAUGGAGGGUUUCAAU
siCDC37-2 antisense	AUUGAAACCCUCCAUGUAC
siE2F1-1 sense	GCGGAGGCUGGACCUGGAA
siE2F1-1 antisense	UUCCAGGUCCAGCCUCCGC
siE2F1-2 sense	CAAGGCCCGAUCGAUGUUU
siE2F1-2 antisense	AAACAUCGAUCGGGCCUUG

Primer information

Name	Sequence
AURKB-F	AGGAGAACTCCTACCCCTGG
AURKB-R	AGATGGGGTGACAGGCTCTT
CDC37-F	AACACAAGACCTTCGTGGAAAA
CDC37-R	TAATTGGCTGTCTCCTCGCAC
E2F1-F	CATCAGTACCTGGCCGAGAG
E2F1-R	TGGTGGTCAGATTCAGTGAGG
CCND1-F	CAATGACCCCGCACGATTTC
CCND1-R	CATGGAGGGCGGATTGGAA
GAPDH-F	GGAGCGAGATCCCTCCAAAAT
GAPDH-R	GGCTGTTGTCATACTTCTCATGG
ChIP-PCR-AURKB-1-F	CGTCCCTACCTCCTTCCAGC
ChIP-PCR-AURKB-1-R	GCGTGGCAGATTCAGTTGTTT
ChIP-PCR-AURKB-2-F	TCGTCGCCCATGCCTAGTTC
ChIP-PCR-AURKB-2-R	CCCTTCTCATTCCGCCTCTTC
ChIP-PCR-AURKB-3-F	GGGTCCAAGGCACTGCTACT
ChIP-PCR-AURKB-3-R	TCACAGGACATCGAGCCAAT

Antibodies and inhibitors information in this work

Antibody	Company	Catalog No
β-actin	abways	AB0035
AURKB	Abcam	ab2254
CDC37	Proteintech	10218-1-AP
p-S13 CDC37	HUABIO	ET1612-74
E2F1	Cell signaling technology	#3742
MYC	Proteintech	10828-1-AP
p-T58 MYC	ZENBIO	R381186
N-cadherin	Proteintech	22018-1-AP
MMP2	Proteintech	10373-2-AP
PARP	Proteintech	13371-1-AP
Caspase 7	GeneTex	GTX123679
Cyclin B1	Proteintech	55004-1-AP
CDK1	Proteintech	19532-1-AP
Flag	Proteintech	66008-4-Ig
His	Proteintech	66005-1-Ig
V5	Proteintech	14440-1-AP
H3	Proteintech	17168-1-AP
p-S10 H3	Proteintech	66863-1-Ig
Bax	Proteintech	0599-2-Ig
Bcl-2	Proteintech	12789-1-AP
Cyclin D1	Proteintech	60186-1-Ig
CDK4	Proteintech	11026-1-AP
Rb	SANTA CRUZ	sc-73598
p-S780 Rb	ZENBIO	R25557
p-S807 Rb	ZENBIO	R381059
Rabbit IgG	Proteintech	B900610
Goat Anti-Rabbit IgG (H+L) HRP	Abways	AB0101
Goat Anti-Mouse IgG (H+L) HRP	Abways	AB0102
Rabbit Anti-Mouse IgG Kappa Light Chain	Proteintech	SA00001-19
Mouse Anti-Rabbit IgG, Light Chain	Proteintech	SA00001-7L
Goat Anti-Rabbit IgG (H+L) Alexa Fluor 594	Abways	AB0518
Goat Anti-Mouse IgG (H+L) Alexa Fluor 488	Abways	AB0142
Cycloheximide (CHX)	MCE	HY-12320
MG132	MCE	HY-13259
AZD1152-HQPA	Selleck	S1147

Original western blot of Fig 2

2B

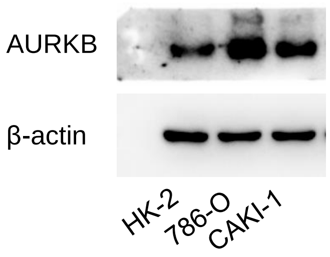

2I

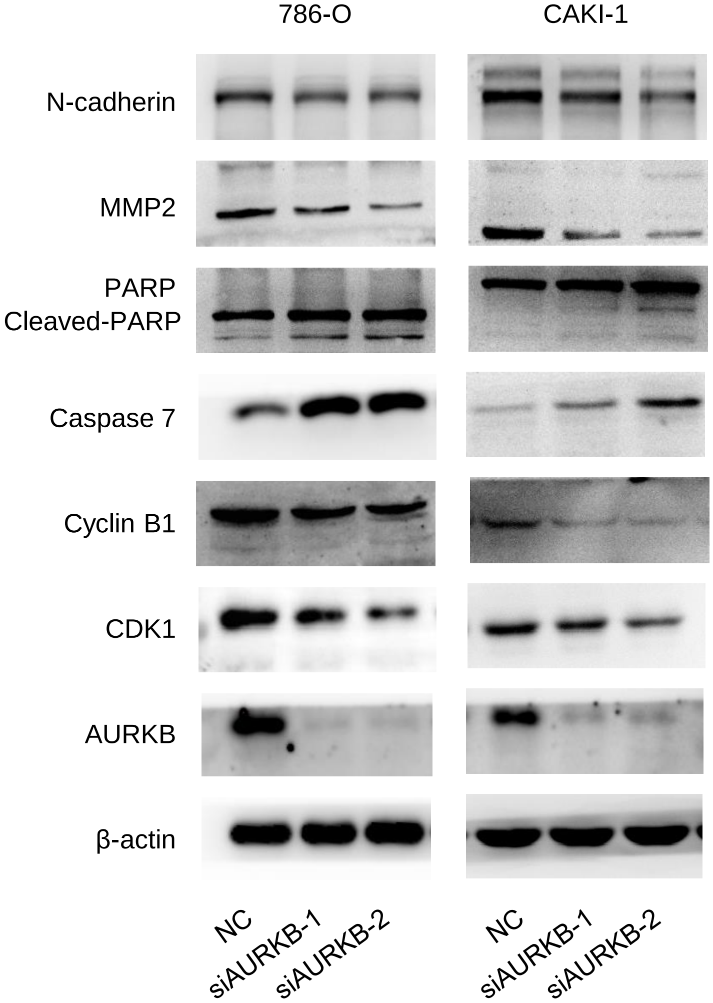

2M

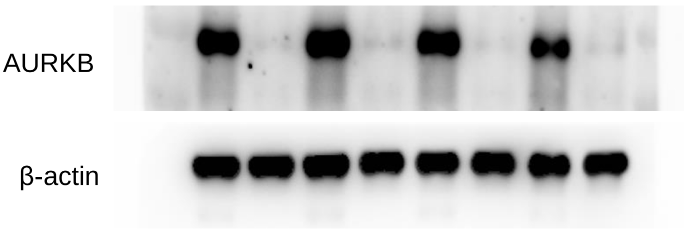

Original western blot of Fig 3

3B

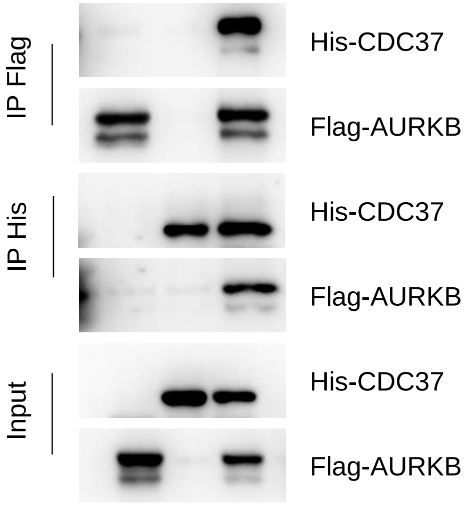

3D

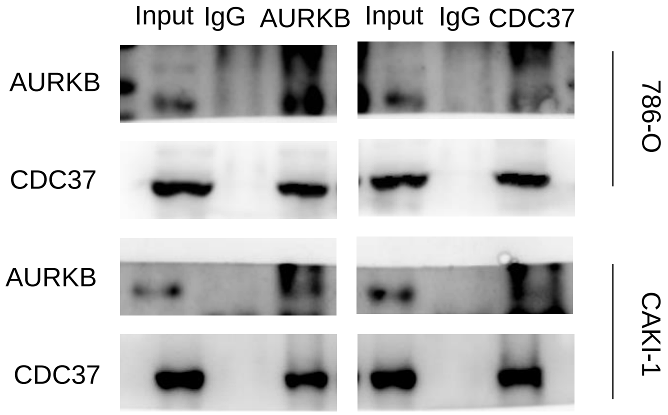

3E

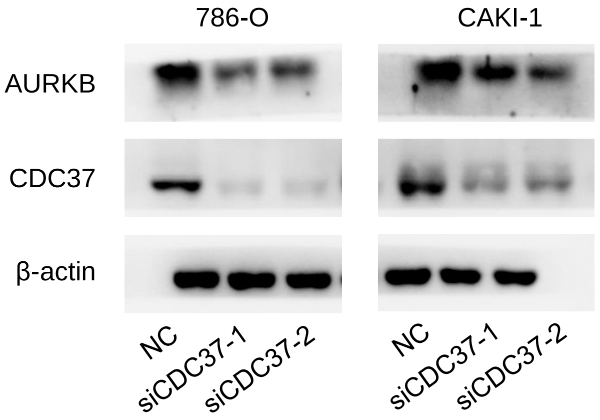

3F

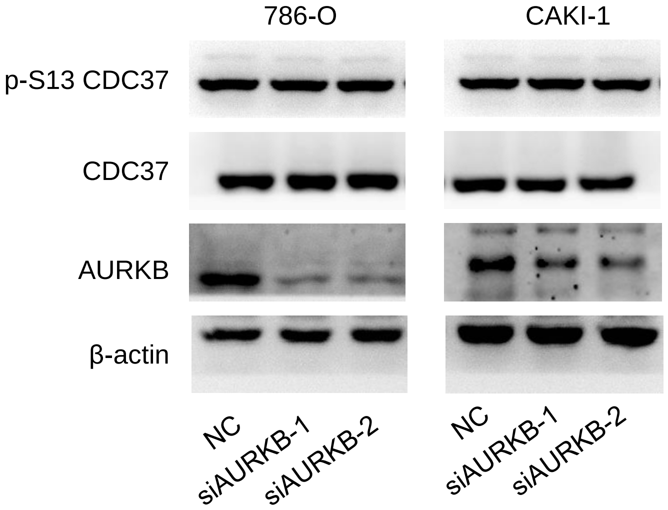

Original western blot of Fig 4

4A

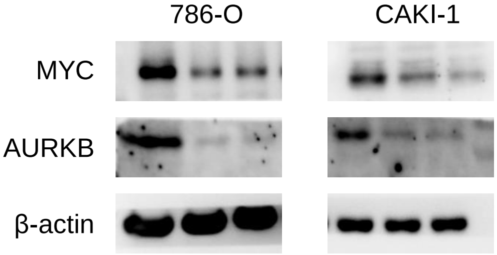

4B

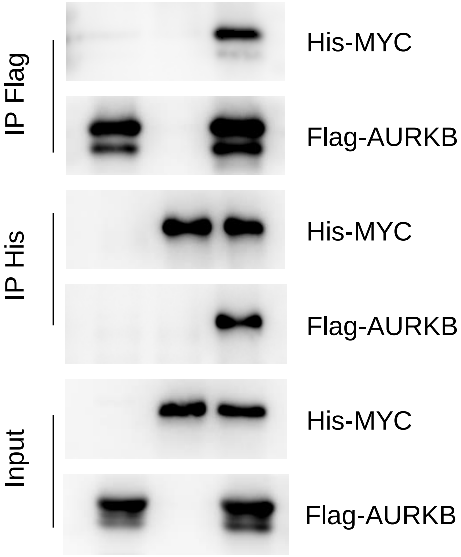

4D

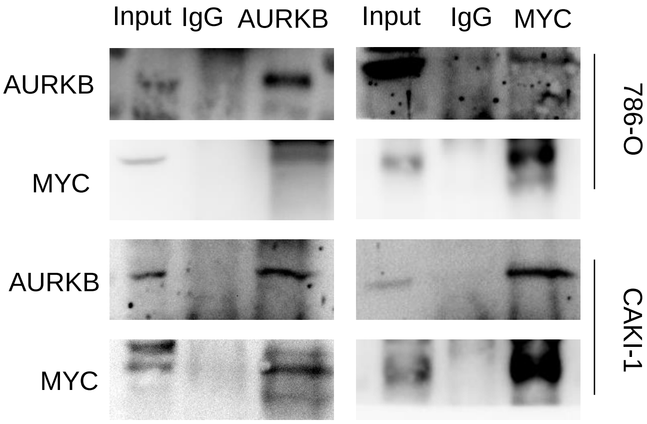

4E

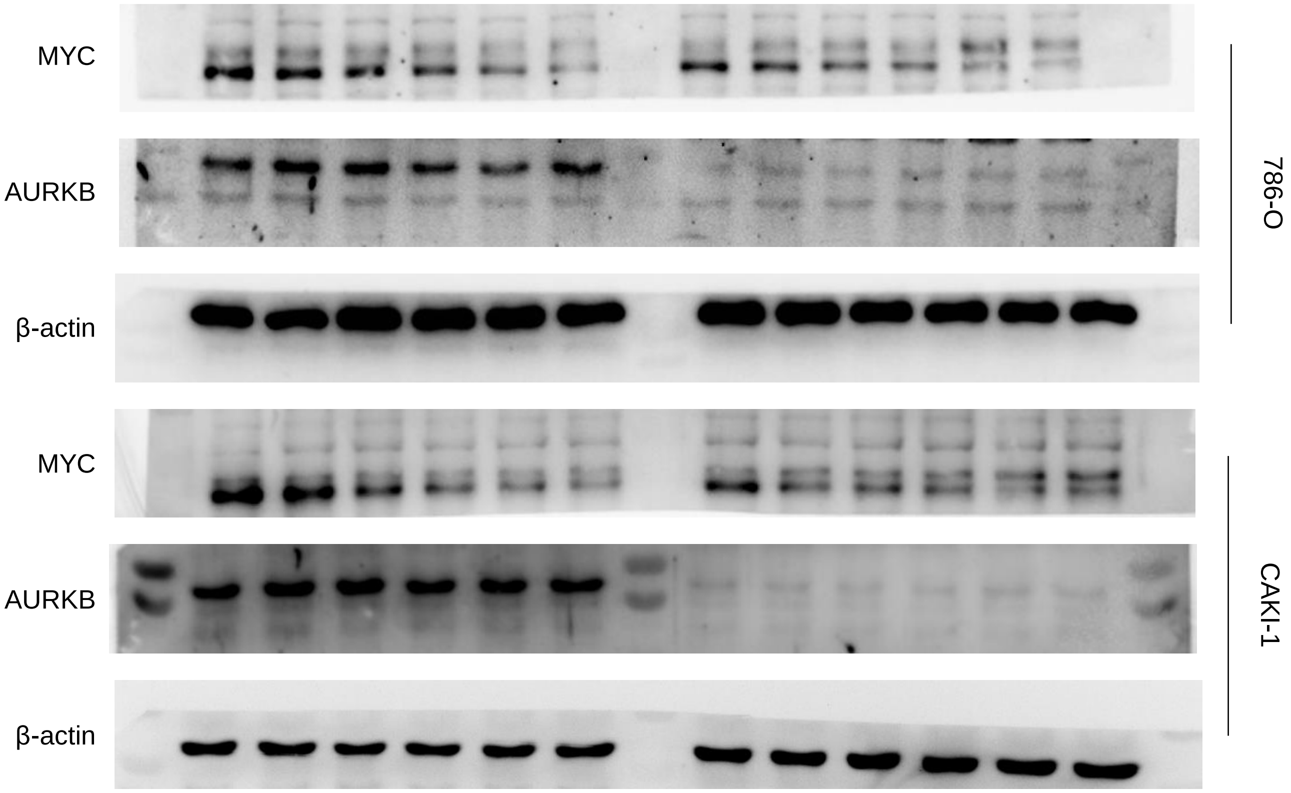

Original western blot of Fig 4

4F

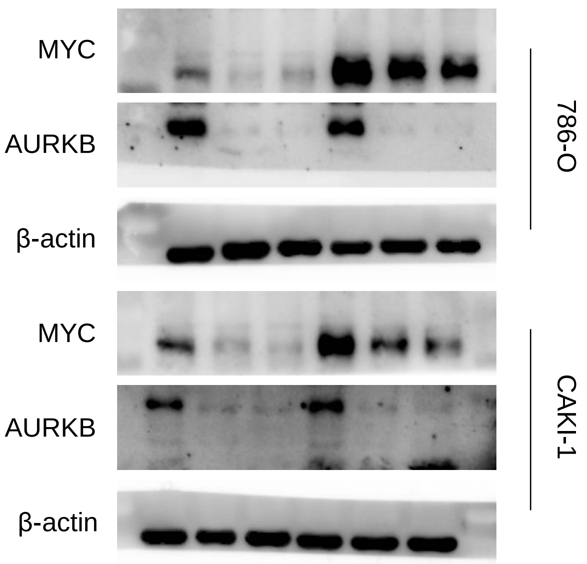

4G

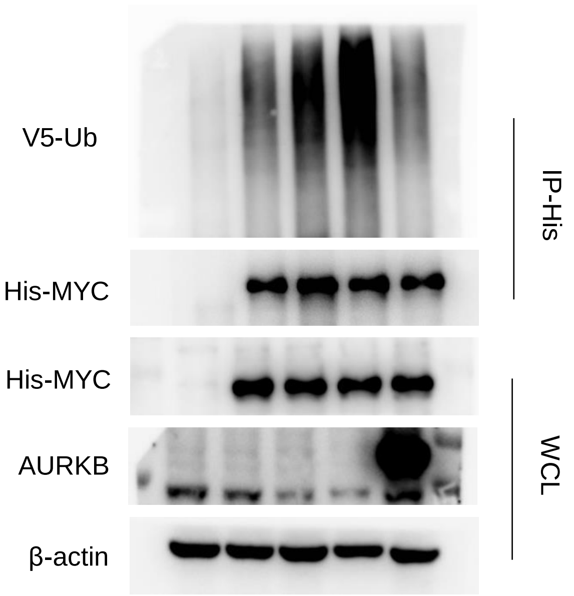

4H

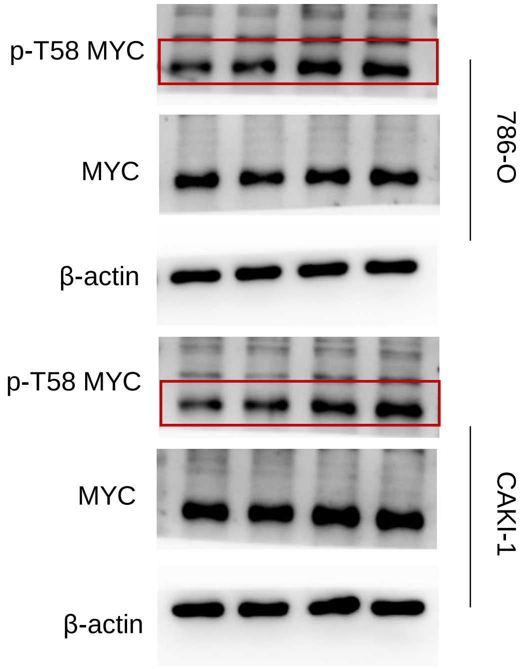

4I

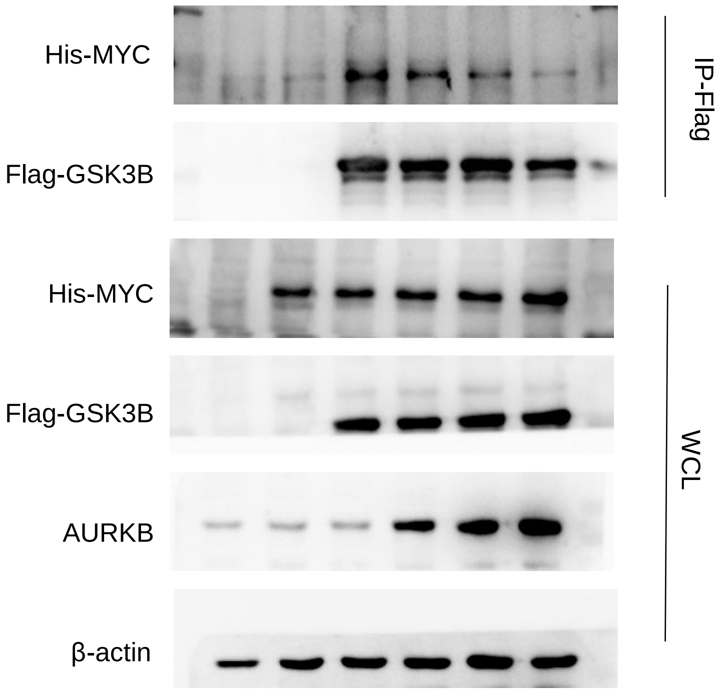

4J

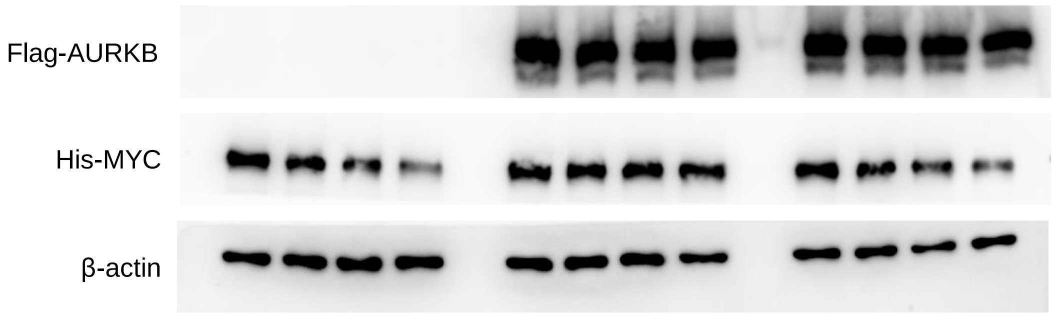

Original western blot of Fig 4

4L

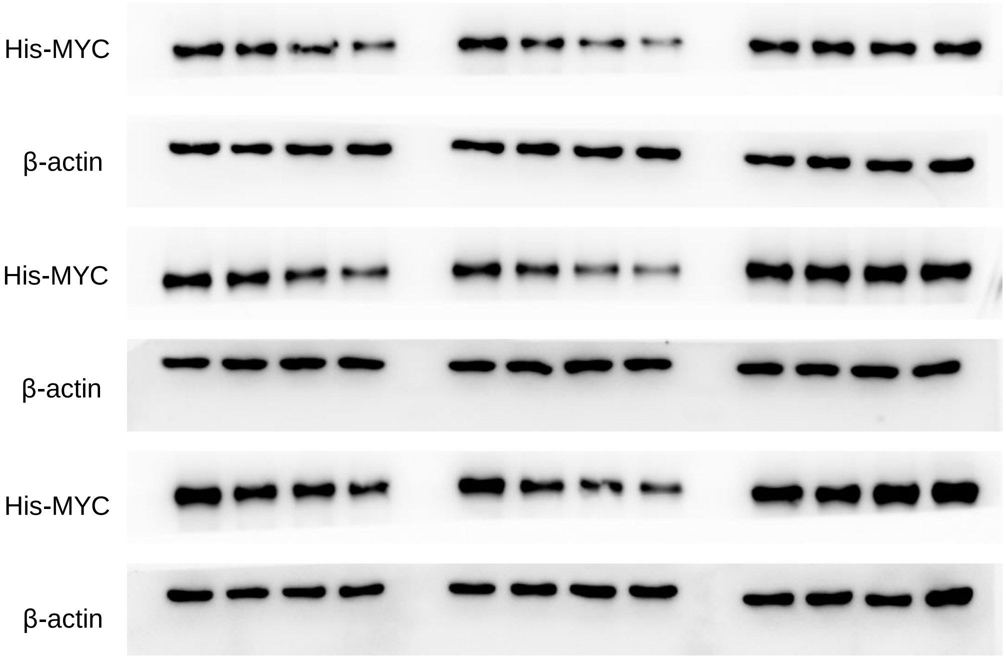

4M

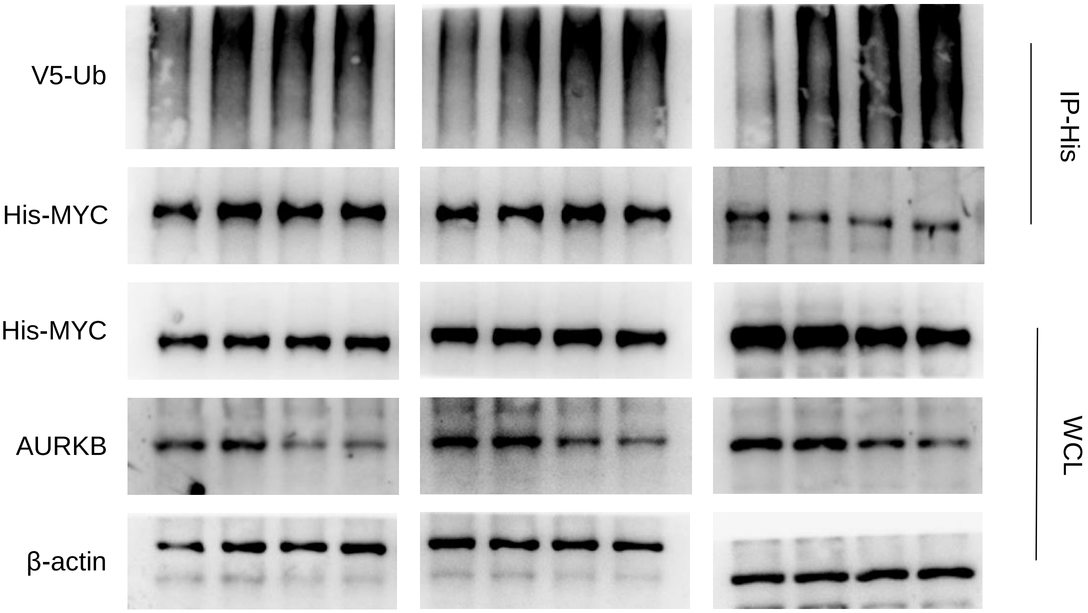

4N

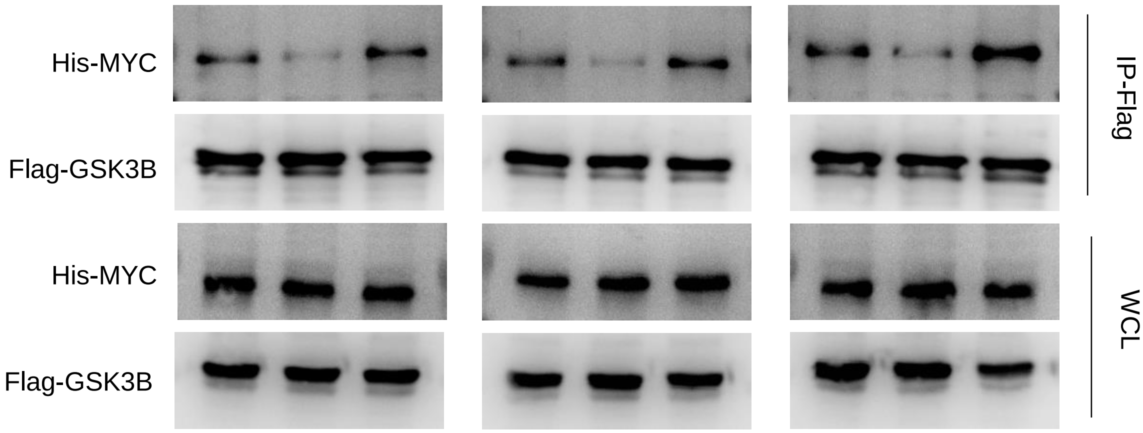

Original western blot of Fig 5

5A

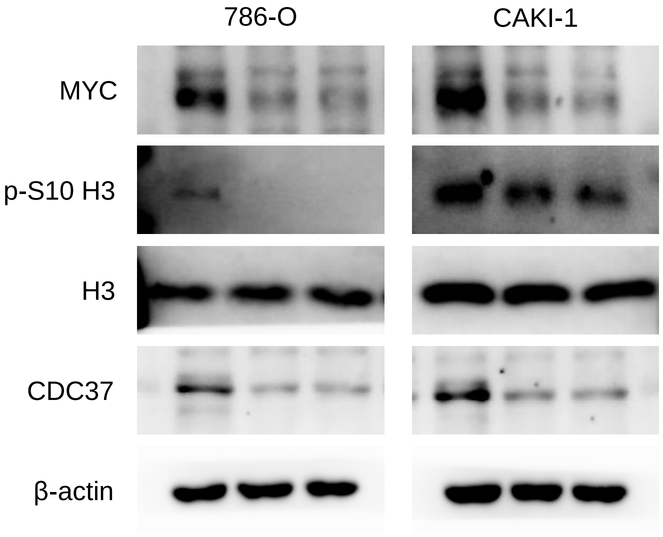

5C

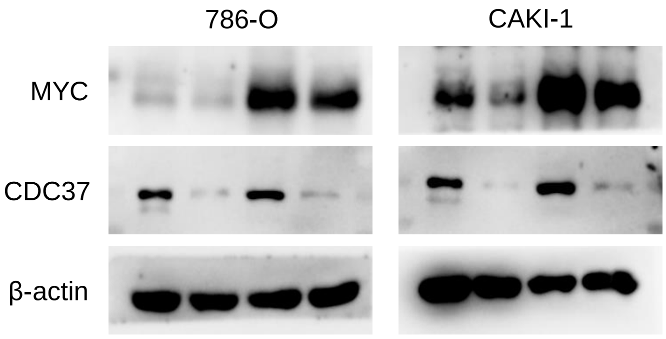

5B

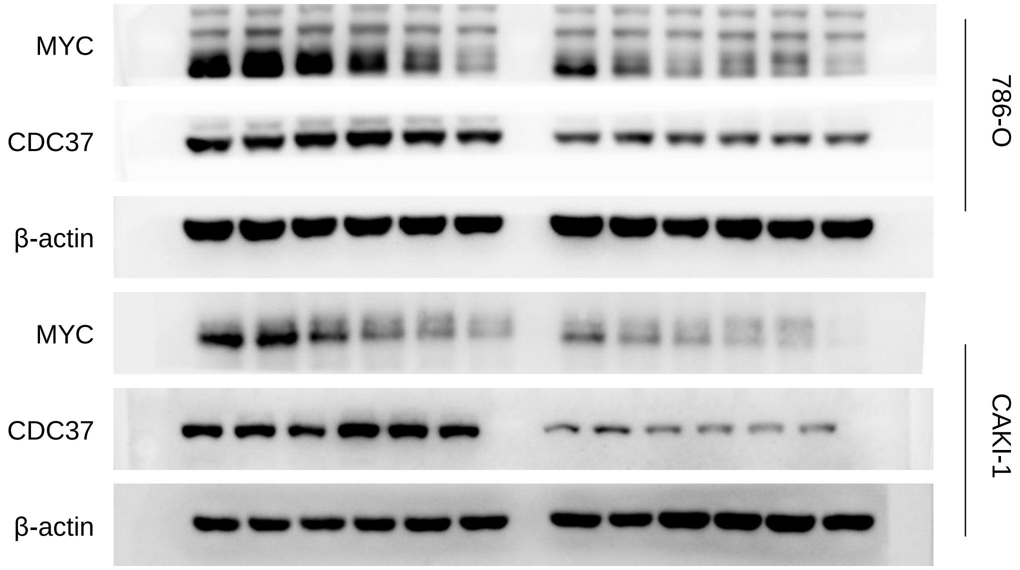

5D

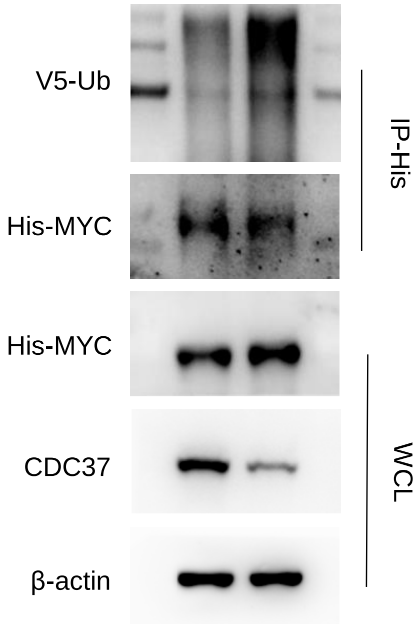

5E

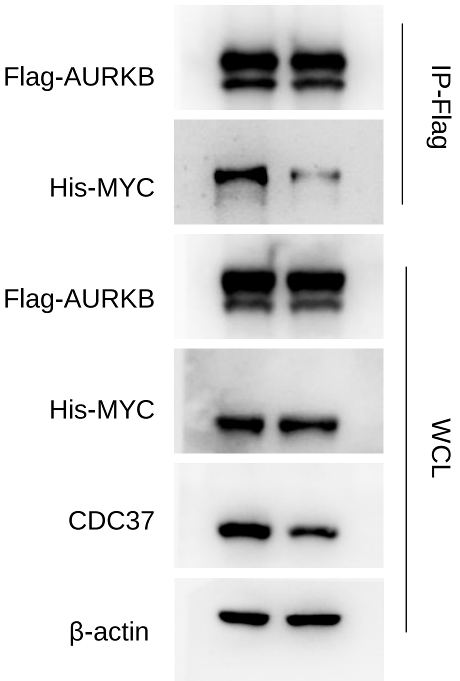

Original western blot of Fig 7

7E

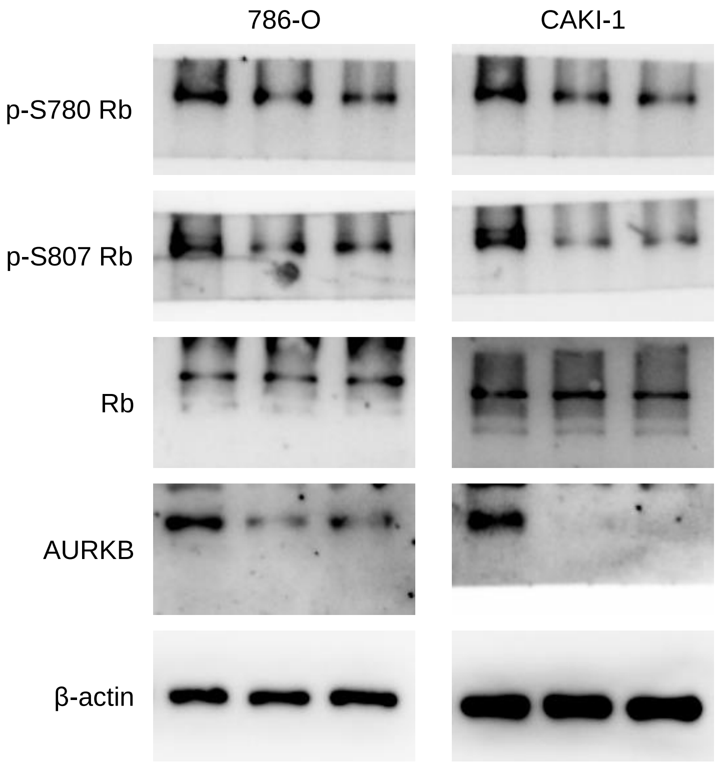

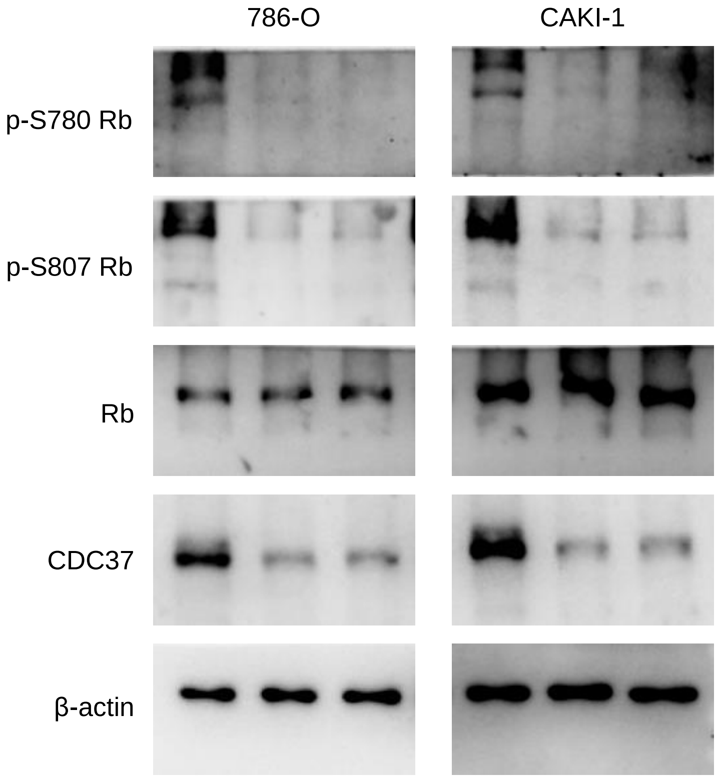

Original western blot of Fig S

S4D

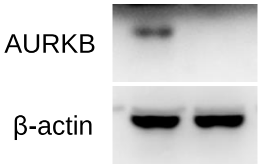

S5I

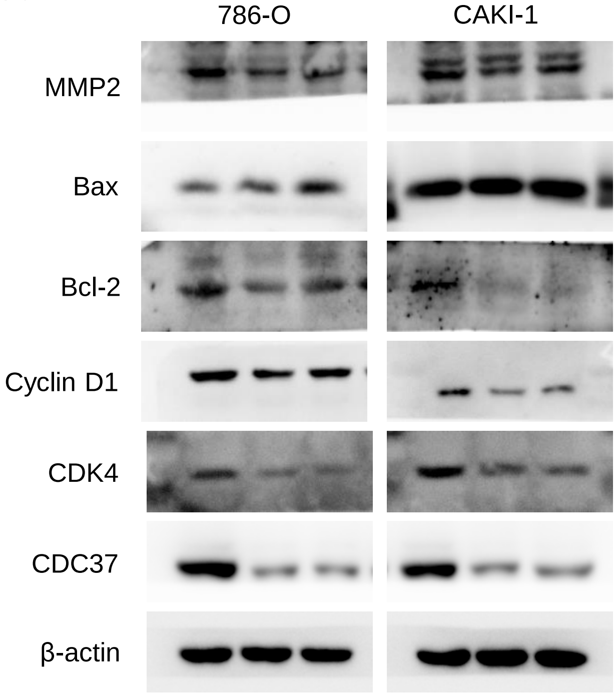

S6A

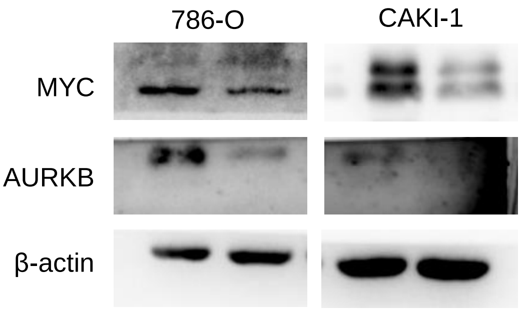

S6B

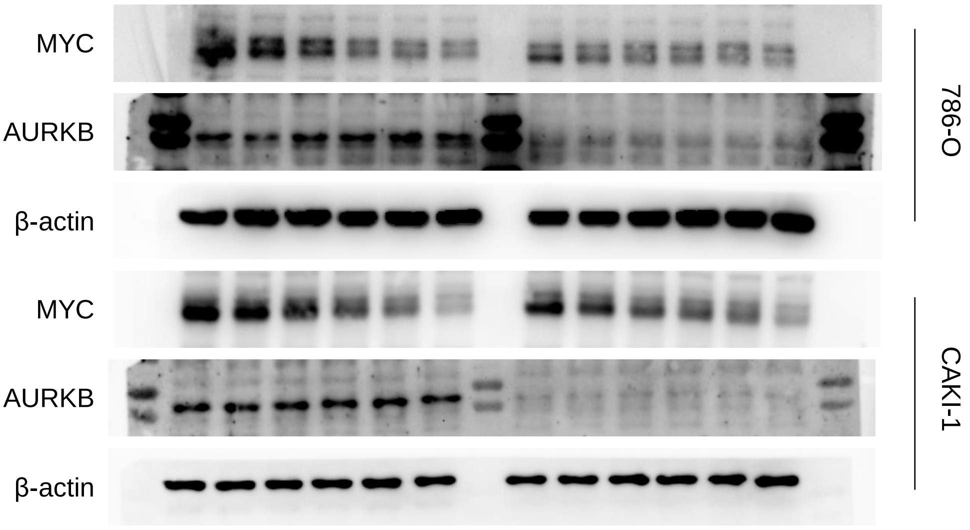

S6C

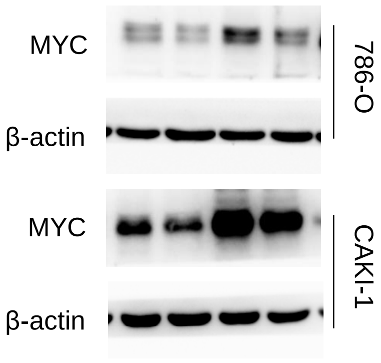

S6D

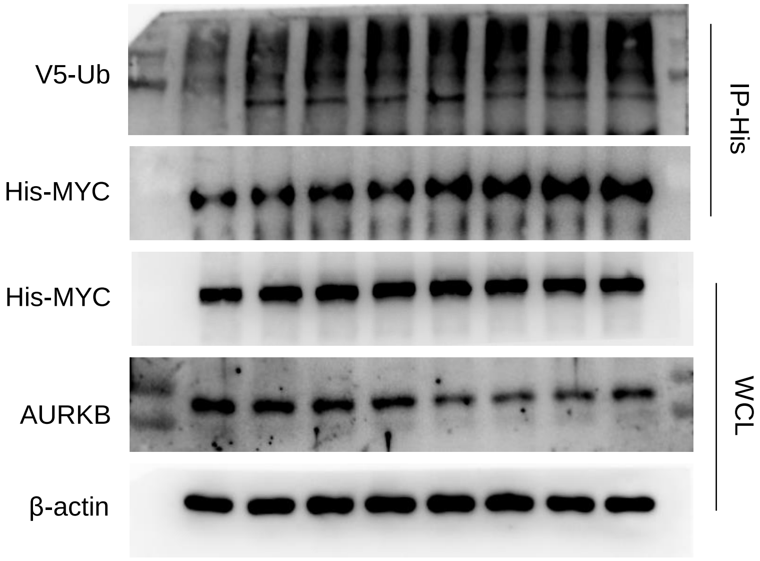

Original western blot of Fig S

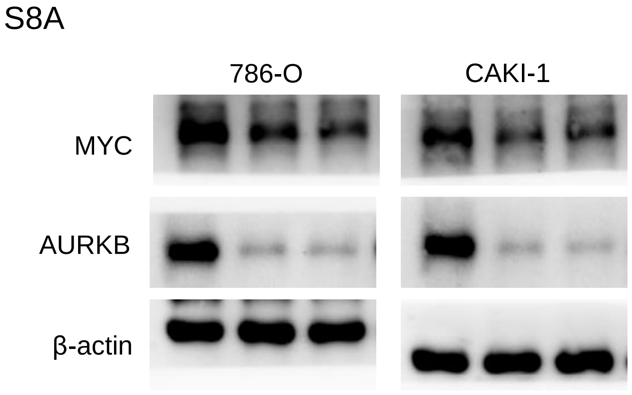

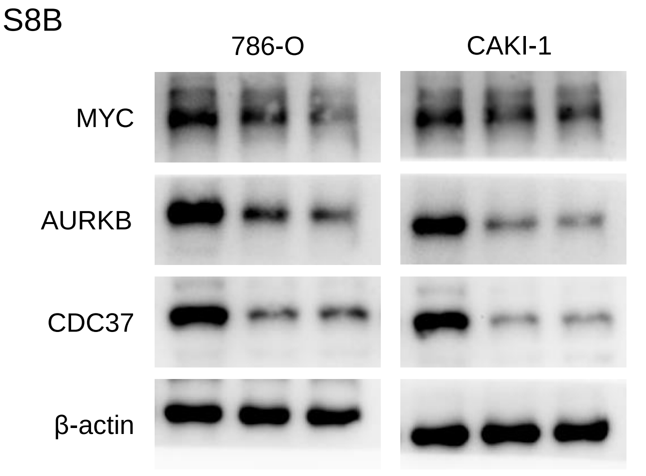

Supplement: Supplementary file 1 — Supplementary information [file 41419_2024_6827_MOESM1_ESM.pdf]
